# Dynamics of oral human papillomavirus infection in healthy population and head and neck cancer

**DOI:** 10.1002/cam4.5686

**Published:** 2023-02-27

**Authors:** N. V. Vani, R. Madhanagopal, R. Swaminathan, T. S. Ganesan

**Affiliations:** ^1^ Epidemiology, Biostatistics, and Tumour Registry Cancer Institute (WIA) Chennai India; ^2^ Medical Oncology Cancer Institute (WIA) Chennai India; ^3^ Present address: Department of Medical Oncology Sri Ramachandra Institute of Higher Education and Research Chennai India

**Keywords:** dynamics, head and neck cancer, human papillomavirus, oral infection, prevention

## Abstract

The recent increase in high‐risk human papillomavirus (HR‐HPV)‐associated oral and oropharyngeal cancers has gained considerable importance due to their distinct clinical and molecular characteristics. However, the natural history of oral HPV from acquisition to persistence and malignant transformation is still unclear. The global prevalence of oral HPV infection in healthy individuals ranges from 0.67% to 35%, while 31%–38.5% in head and neck cancer (HNC). The persistence rate of oral HR‐HPV infection is 5.5% –12.8% globally. India has the highest HNC burden due to apparent differences in predisposing factors compared with the West. The prevalence of oral HPV in healthy individuals and its contribution to HNC is less evident in Indian studies. HR‐HPV‐associated HNC in this region accounts for 26%, with an active infection in 8%–15% of these tumors. There is a lack of concordance in the expression of p16 as a surrogate marker for HPV detection in HNC because of differences in behavioral risk factors. Due to a lack of evidence, treatment de‐escalation cannot be implemented despite the improved outcome of HPV‐associated oropharyngeal cancers. This review critically analyzes the existing literature on the dynamics of oral HPV infection and HPV‐associated HNC, identifying potential avenues for future research. A better understanding of the oncogenic role of HR‐HPV in HNC will help to formulate novel therapeutic approaches and is expected to have a significant public health impact as preventive strategies can be implemented.

## Graphical abstract

High‐risk human papillomavirus (HR‐HPV)‐associated oral and oropharyngeal cancer has recently gained clinical attention due to their distinct clinical and molecular characteristics. However, the natural history of oral HPV from acquisition to persistence and malignant transformation is still unclear. This review critically analyzes the literature on the dynamics of oral HPV infection and HPV‐associated head and neck cancer (HNC), identifying potential avenues for future research.

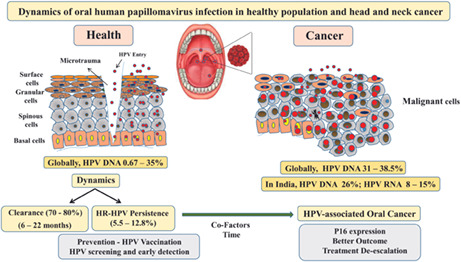

## 1 INTRODUCTION

The global incidence of head and neck cancer (HNC) is about 660,000 cases, with a mortality of 325,000 patients annually.^1^ Tobacco consumption (smoking and smokeless) has contributed to an increased incidence of HNC (6.1 per 100,000 women and 20.9 per 100,000 men) in the Indian subcontinent, accounting for one‐third of the global burden.^2^ The recent increase in HNC in nonsmoking, nonalcoholic young individuals is primarily attributed to mucosal high‐risk human papillomavirus (HR‐HPV) infection.^3^ Though Zur Hausen reported the oncogenic role of HPV in cervical cancer in 1974, its role in HNC was identified later by Syrjanen.^4^ Extensive research has proven that HR‐HPV‐associated HNC is a distinct clinical entity that differs substantially from those caused by traditional risk factors, for example, smoking, in all aspects—genetic, molecular, epidemiological, and clinical. This review critically analyzes the existing literature on the dynamics of oral HPV infection and HPV‐associated HNC, identifying potential avenues for future research. It also traces the pathogenesis and the epidemiology of HR‐HPV‐associated HNC globally and in India, which has a higher disease burden and exhibits different behavioral risk factors. HR‐HPV‐associated oropharyngeal cancers (OPSCC) have shown better treatment outcomes due to a distinct mutational profile. A better understanding of the oncogenic role of HR‐HPV in HNC will help to formulate novel therapeutic approaches and is expected to have a significant public health impact as preventive strategies can be implemented.

## 2 PATHOPHYSIOLOGY OF HR‐HPV‐ASSOCIATED HNC

Human papillomaviruses are double‐stranded, circular DNA viruses that infect basal epithelial cells through microtrauma. Most HPV infections are transient and cleared by the host immune system without clinical symptoms. Both high‐risk and low‐risk‐HPV (LR‐HPV) exhibit restricted viral gene expression sufficient for basal cell genome maintenance and persistence.^5^ Nevertheless, HR‐HPV E6/E7 proteins have the potential to induce carcinogenesis, as they have complex immune evasion functions and can suppress cytokine signaling and the presentation of antigens to dendritic cells. The LR‐HPV have a poor ability to induce neoplasia due to different transcriptional regulation of E6/E7 genes, and their proteins do not induce extensive cell proliferation in the basal and parabasal cell layers. However, host genetic susceptibility and immune suppression might lead to the persistence of both HR‐HPV and LR‐HPV, and a deregulated HPV gene expression leads to malignant transformation.^6^

Integration of the HPV genome into human chromosomes and expression of E6/ E7 oncoproteins have been the classic mechanism for HPV‐mediated oncogenesis. However, extrachromosomal circular oncogenic DNA (ecDNA), including viral circular DNA (vcDNA), human ecDNA, or viral–human hybrid ecDNA, was observed as a dominant feature of HPV+ (HPV‐positive) OPSCC.^7^ The progression of benign HPV infection to HPV‐associated OPSCC requires the maintenance of stable ecDNA or vcDNA as part of carcinogenesis. The three primary viral genomic forms observed in HPV+ OPSCC are—ecDNA (episomal form) with a broad expression of HPV gene products; integrated form with an expression of HPV gene products and overexpression of human hybrid transcripts; and concomitant form (episomal and integrated forms) with overexpression of E6/ E7 with a dramatic reduction in early E2, E4, and E5 HPV gene product expression.^7^ The cancer genome sequencing data have shown that 73% of HPV16+ HNC with high copy numbers had an episomal form of the viral genome that is likely to replicate in an E1–E2‐dependent manner.^8^ Recently, whole‐genome sequencing and optical genome mapping of HPV+ OPSCCs have shown integrated viral forms, including concomitant forms (episomal and integrated forms) in two‐thirds of the tumors. This viral integration correlated with pervasive genome‐wide somatic alterations at sites distinct from the integration site. However, few or no somatic mutations were present in tumors with only episomal HPV.^9^ Multiple HPV integration sites are often seen in a single tumor. Still, only a few are transcriptionally active, while others can be considered silent passenger integrants. Integration frequently occurs in a genetically unstable environment, but only those integration events that provide cells with a selective growth advantage drive clonal expansion and oncogenesis.^10^ The mere presence of the virus alone does not determine the viral activity (transforming infection). The latter is established by viral load and deregulated E6/E7 mRNA expression.

HPV viral loads are lower in HNC, except in OPSCC, which exhibits higher loads, mainly in the episomal form.^11^ These viral loads are usually determined by dividing the HPV DNA copy number by the total number of cells, estimated through quantification of β‐globin by real‐time PCR. Increased HPV viral load indicates viral shedding and an increased risk for HPV transmission and persistence. It also leads to increased expression of E6/E7 oncoproteins, suppressing p53 and pRb, and indirectly causing p16 overexpression.^12^ High HPV viral loads have been shown to upregulate MHC class I after radiation, which improves immunosurveillance of residual disease and hence better survival.^12^ p16 overexpression is an excellent biomarker for HPV‐associated malignancies.^13^ Usually, p16 is lost or silenced in most malignancies, allowing cell cycle progression. However, overexpression of p16 (in response to E7 oncoprotein) is triggered by a cellular senescence response mediated by H3K27‐specific demethylase (KDM)6B. HPV E7 causes an acute dependence on KDM6B expression for cell viability. The p16INK4A tumor suppressor is a critical KDM6B downstream transcriptional target, and their oncogenic activity depends on the inhibition of CDK4/CDK6. The biological activity of p16 in HPV‐associated cancers is more that of an oncogene than its well‐established tumor‐suppressor activity in most other human tumor types.^14^

A recent study has reported an intact E2 gene in 68% of HPV16+ OPSCC along with a high viral load and E2/E6/E7 oncogene expression with improved clinical outcomes compared with patients with disrupted E2 gene.^15^ The presence of intact E2 gene in HPV+ OPSCC is associated with methylation of E2 binding sites (E2BS3 and E2BSx4) in the long control region (LCR), leading to loss of protein expression, thereby creating the same effect as E2 deleted gene.^16^ Studies have shown that E2 disruption upon viral integration will not per se lead to increased expression of E6/ E7 oncogenes, suggesting that constitutive rather than a high‐level expression of viral oncogene transcripts is required in HPV‐induced carcinogenesis.^17^ E5, a small oncoprotein, exhibits cell transforming activity by increasing epidermal growth factor receptor (EGFR) and Met (HGF) receptor expression, thereby enhancing growth factor signal transduction.^18^ The E5 oncogene is variably expressed in cervical cancer, as it is deleted during viral integration. However, E5‐encoded transcripts vary and correlate with EGFR expression in HPV‐associated OPSCC.^19^ A decreased EGFR expression was reported along with increased E5/E6/E7, probably due to EGFR methylation.^20^ The exact role of E5 in head and neck carcinogenesis and its correlation with EGFR needs further research.

## 3 GENETIC AND EPIGENETIC ALTERATIONS IN HR‐HPV‐ASSOCIATED HNC

The virus‐host interactions modulate the host genetic and epigenetic landscape that facilitates tumorigenesis. HPV‐positive tumors have exhibited a complete mutual exclusivity with driver mutations in TP53, CDKN2A, and TERT. HPV is associated with an increase in mutations attributed to the mutational signature 2, as evidenced by the overexpression of APOBEC3B.^21^ Mutations in the oncogene PIK3CA with an increase in PI3K‐AKT‐mTOR signaling, loss of TRAF3, and amplification of E2F1.^22^ Understanding the cellular alterations of HPV‐related HNC help to devise targeted therapy and identify patients for treatment de‐escalation.

Epigenetic alterations like DNA hypermethylation are common in HPV‐related OPSCC. Checking for DNA methylation of a group of cellular or viral genes has been proposed as a second triage in cervical cancer screening programs. It helps to identify the premalignant lesions that could potentially progress (persistent infection) from transient infection.^23^ Hypermethylation of 20 highly specific genes (KCNA3, EMBP1, CCDC181, DPP4, ITGA4, BEND4, CTNND2, ELMO1, SFMBT2, C1QL3, MIR129–2, ATP5EP2, OR6S1, NID2, HOXB4, ZNF439, ZNF93, VSTM2B, ZNF137P, and ZNF773.) were found in HPV+ OPSCC samples, which can be used for detection and disease management.^24^ The EPB41L3, L1, L2, and E2 (HPV16) methylation marker panel has been evaluated for the early diagnosis of OPSCC. It has shown 70% sensitivity and 91% specificity and can be done in a simple oral gargle sample.^25^ The dynamic and reversible nature of epigenetic modifications makes them ideal therapeutic targets for treating HPV‐associated cancers.

Viral miRNAs modulate cellular and viral gene expression, promoting carcinogenesis and evading the host's immune system. HPV16 encodes HPV16‐miR‐1, HPV16‐miR‐2, and HPV16‐miR‐3, which are known to target cellular genes promoting carcinogenesis.^26^ miRNA expression profiling study revealed that miR‐127‐3p, miR‐363, miR‐20a, miR‐34a, let‐7c‐5p, and miR‐9 are found predominantly in HPV+ oral cancer (OSCC).^27^ HPV‐induced epigenetic alterations, viral noncoding RNAs, and methylation signatures could serve as potential biomarkers and therapeutic targets for new antiviral drugs.

## 4 HPV SCREENING AND DETECTION TECHNIQUES

Currently, the HPV tests approved by the United States Food and Drug Administration (FDA) are those intended for use in cervical cancer. There are no HPV diagnostic tests with regulatory approval for OPSCC. HPV testing options available for use in the clinical setting for HNC are laboratory‐developed tests (LDTs), which involve various techniques and platforms for the analysis of HPV DNA, HPV messenger RNA (mRNA), or the p16 protein.^28^ The enormous number of assays used for HPV detection has been developed mainly for cervical cancer. Primarily, they detect HPV infection at the DNA level with or without genotyping information. Molecular techniques like signal amplification assays (DNA/RNA ISH) and high‐throughput target amplification assays (HPV DNA PCR, qPCR, and RT‐PCR) are used principally for HPV detection^29^ Testing for HPV RNA is essential for the molecular diagnosis of clinically relevant HPV infections, as it evaluates the gene expression indicating an active infection. HPV‐16 E6/E7 transcripts can be detected by reverse transcriptase PCR (RT‐PCR) and qRT‐PCR. Gene expression by microarray has recently gained acceptance as a high‐throughput method.^30^ Droplet digital PCR (DD‐PCR), the new generation HPV assay, is a highly sensitive method for the quantitative and qualitative detection of HR‐HPV with high precision and reproducibility.^31^ DD‐PCR can also be employed for detecting circulating tumor DNA (ctDNA) and screening and monitoring response to treatment.^32^ HPVctDNA and E6 humoral response are performed on blood and, therefore, noninvasive. Baseline E6 humoral levels have a prognostic value, and HPVct DNA helps to monitor HPV+ OPSCC recurrence.

Next‐generation sequencing (NGS)‐based “capture HPV” is feasible on biopsies and ctDNA. It helps characterize HPV integration status and sites and could define prognostic subgroups in HPV+ OPSCC.^33^ HPV‐Seq using dual‐strand full‐length viral capture enables the detection of less than one copy of ctDNA. It also allows the identification of HPV genotype, mapping location along the HPV genome, and ctDNA fragment length distribution. It holds clinical implications for treatment monitoring of HPV‐related cancers, including early detection and minimal residual disease (MRD) testing.^34^ HPV pseudovirion‐based neutralization assay (PBNA), competitive or total Luminex immunoassays (cLIA or LIA), and virus‐like particle (*VLP*) based enzyme‐linked immunosorbent assays (ELISA) are the standard serological assays used to measure the HPV‐specific antibody responses following vaccination.^35^

The widely used test in clinical practice is p16 (INK4A) detection by immunohistochemistry (IHC). p16 protein is a surrogate marker of transcriptionally active HR‐HPV infection with a sensitivity of 100%. It has demonstrated good agreement with HPV E6/E7 mRNA expression detected by RT‐PCR and RNA ISH.^36^ It is inexpensive and can be performed on formalin‐fixed paraffin‐embedded tissue (FFPE) samples. In the modified 8th AJCC/UICC classification, p16 was recommended as an HPV surrogate marker in OPSCC if there was diffuse (≥75%) expression and at least moderate (+2/3) staining intensity in the tumor.^37^ The various methods described for HPV detection are used only in research, whereas in clinical practice, it is based on p16 detection by immunohistochemistry. But the role of p16‐IHC for HPV detection in HNC is still a matter of debate, as many studies have found discordance. This is apparent in Indian patients due to p16 hypermethylation driven by prolonged smokeless tobacco exposure.^38^ p16 expression in HPV‐negative tumors is due to transcriptional upregulation by factors such as Ets and Myc, alterations of Ras‐MAPK pathways, and loss of Rb.^39^ In addition, such tumors exhibit *TP53* mutations and high levels of EGFR expression, which is absent in HPV+ tumors.^40^ Here, p16 expression reflects an entirely different oncogenic process that improves prognosis by inducing a senescent phenotype that slows tumor growth.^39^ However, double positivity for HPV DNA/p16 has the strongest diagnostic accuracy and prognostic value for HPV‐related OPSCC.^41^ Therefore, p16 is an effective surrogate marker for HPV detection in OPSCC and must be confirmed by HPV DNA to consider causality.

## 5 PREVALENCE AND NATURAL HISTORY OF ORAL HPV INFECTION IN HEALTHY INDIVIDUALS

The average global prevalence of oral mucosal HPV infection in healthy individuals is 4.9% (0%–24%).^42^ Higher prevalence was reported in Europe (6.5%), followed by North America (5.1%), Latin America (4.6%), and Asia (3.1%), most likely due to different sexual behaviors and tobacco consumption.^42^ A US population‐based survey reported an oral HPV prevalence rate of 7% (HR‐HPV 3.5%); however, the lifetime risk of developing HPV‐associated OPSCC was lower (37 per 10,000) among those with oral HR‐HPV infection.^43^ Cutaneous HPV types (5.46%) were predominant compared with mucosal types (0.67%) in the Chinese population.^44^ The prevalence of oral HPV infection in healthy individuals globally from all published reports ranges from 0.67 to 35% (Table 1) (Supplementary I). The Australian indigenous population had the highest prevalence rate of 35.3%, with a wide genotype distribution.^45^ In a report evaluating homosexual males, 10.6% had oral HPV infection by DNA PCR, while only 12.9% of them had detectable E6/E7 mRNA, suggesting active infection.^46^ The prevalence of oral HPV was 23.7% (any HPV) and 2.4% (HR‐HPV) in Indian HIV‐positive men who have sex with men (MSM).^47^ But, data on the prevalence of oral HPV infection in healthy individuals in the Indian population are unavailable.

**TABLE 1:** Oral HPV (DNA) prevalence in healthy individuals.^a^

References	Study group	Sample size	Primers / genotyping	HPV positivity	Genotypes
Hang et al. China^44^	General population	5351	SPF1/GP6+ Sequencing	0∙67% (mucosal types); 5∙46% (cutaneous types)	6,11,16,40,45,58,67,81,90 / 3,10,57
Gillison et al. USA^68^	General population NHANES	4846	Linear array HPV genotyping assay	7∙30%	6,11,16,18,26,31,33,35,39,40,42,45,51–56, 58,58,61,62,66‐73,81–84, CP6 108, IS39
Bettampadi USA Brazil Mexico^110^	HPV infection in men (HIM) study	3098	SPF10 PCR‐DEIA‐LiPA25	7% 8∙7% 10%	16,18,31,33,35, 39,45, 51,52,56,58,59,6, 43,44,53,66,70,74,11,68, 54
Kreimer et al. USA/Mexico/ Brazil^111^	Healthy volunteers (HIM)	1680	Linear array HPV genotyping assay	4%	6,11,16,31,35,39,51,52,53,55,56,58,59,61,62,64,66,69,70,71,72,82,84,89
Pickard et al. USA^112^	University students	1000	Linear array HPV genotyping assay	2∙40%	11,16,35,39,42,45,51,53,55,56,59,62,66,71,72,81,82,84,89
Rosen et al. Peru^113^	General population	980	Linear array HPV genotyping assay	7∙35%	16,18,31,35,39,45,51,52,53,55,56,58,59,61,62,66,71,73,81,83,84
Jamieson et al. Australia^45^	Indigenous Australians	910	MY09/11; GP5+/6+ Nested sequencing	35∙30%	3,6,7,10,13,16,18,30–35, 39,40,42,44,45,51,52,53,54,56,58,59, 62,66,67,68,69,72,73,81,82,84,87,90,10 16/18 (3∙3%), 13/32(22∙8%)

^a^ Search strategy and selection criteria (Supplementary I).

The oral mucosal HPV dynamics from infection to malignancy are still not understood and extrapolated mainly from cervical cancer studies. A study on oral HPV infection (HIM Study) in 1626 healthy men has shown that the mean duration of HPV and HPV16 persistence were 6.9 and 7.3 months, respectively, with clearance of most HPV infections (63%).^48^ Men showed a decreased oral HR‐HPV clearance with age and tobacco smoking.^49^ A systematic review of the dynamics of oral HPV infection has reported a baseline prevalence of 7∙5% (2∙4%–20%), of which HPV16 was 1∙5%. The 12‐month cumulative incidence was 4∙8% (3∙2%–7∙3%), with a median time of 6∙5–18 months for clearance (0%–80%), the longest of which was for HPV‐16 (7–22 months; 43%–83%).^50^ A higher incidence rate of 25% at 24 months was reported in the Australian population, with a baseline prevalence of 10.7%. The clearance rate was 79% at 9 months, and the persistence rate was 20% (any HPV type) and 12.8% (HR‐HPV) at a follow‐up 24 months later. A higher persistence rate in this study may be due to the selected cohorts and the HPV detection method employed.^51^ However, the clearance rate in high‐risk individuals in another report was 71% for prevalent infection and 97% for incident infections, while the persistence of HR‐HPV infections was 5.5% at a 7‐year follow‐up.^52^ Similar data on the dynamics of oral HPV infection among healthy individuals in India is unavailable, underscoring the need for natural history studies.

## 6 PREVALENCE OF HPV‐ASSOCIATED HNC

The average global prevalence of HR‐HPV DNA positivity in HNC is 45·8% (OPSCC), 24·2% (OSCC), and 22.1% (laryngeal cancer).^53^ However, these figures do not reflect true prevalence due to etiological heterogeneity and differences in detection methods. A pooled prevalence of 34∙5% HPV DNA+ HNC was reported, with PCR‐based studies showing a higher prevalence (34∙8%) than ISH‐based studies (32∙9%).^54^ The global prevalence of HR‐HPV infection in HNC varies from 31% to 38∙5% and is almost similar across Asia, Europe, North America, and South America. It was higher in Australia (42∙5%) and lowest in Africa (11∙7%), probably due to a limited number of studies in the latter. The common genotypes observed were HPV16 (74∙7%) and HPV18 (24%) (Table 2) (Supplementary I and II). OPSCC is five times more likely associated with HR‐HPV than oral, laryngeal, and other pharyngeal cancers.^55^ Most studies have determined the presence of HR‐HPV DNA without evaluating the biomarkers of HPV carcinogenesis (i.e., E6/E7mRNA, p16 protein), thus failing to distinguish between transient and persistent HPV infection. OPSCC (39∙8%) had higher E6/E7 HR‐HPV mRNA positivity than OSCC (16∙3%) and laryngeal cancers (8∙6%), thereby strongly suggesting the oncogenic role of HPV in these sites. Tonsils (53·9%) had the highest HPV DNA prevalence among these sites.^56^

**TABLE 2:** Global prevalence of HPV DNA in head and neck cancer.^a^ ^,^ ^b^

Continent	Year, country	No. of studies	No. of cases	Overall HPV prevalence weighted %	Common HR‐HPV genotypes
Asia	1991–2020 Bangladesh, China, Hong Kong, Indonesia, Iran, Israel, Japan, Jordan, Korea, Lebanon, Malaysia, Pakistan, S Korea, S Arabia, Sri Lanka, Taiwan, Thailand, Turkey	71	7261	29∙8	16 (72%); 18 (26%); 33(6%); 52(6%); 58(5%); 35(2.7%)
Africa	1995–2019 Ghana, S. Africa, Sudan, Senegal	11	961	11∙7	16(58%); 18(53%); 33(14∙5%); 31(12%)
Australia	2010–2017 Australia	6	1880	42∙5	16(95%); 18(2%); 33(2%); 35(1%)
Europe	1990–2020 Belgium, Czech Republic, Finland, France, Germany, Greece, Hungary; Italy, Netherlands, Norway, Slovenia, Spain, Sweden, Switzerland, UK	67	10,890	29∙8	16(75∙5%); 18(24%); 33 (7∙6%); 31(8∙4%); 45(7∙8%); 58(7∙4%); 35(5∙8%); 52(5∙4%)
North America	1992–2019 USA, Canada, Puerto Rica	35	4075	37∙8	16(79%); 33(11%); 18(8∙6%); 58(4%); 59(3∙6%); 35(3∙3%)
South America	1994–2021 Argentina, Brazil, Colombia; Mexico, Venezuela	15	927	29	16(76∙8%); 18(32∙5%); 33(7%); 35(4%)
Total		205	25,994	31.3	31∙3%

^a^ References are given in Supplementary II, IV.

^b^ Search strategy and selection criteria (Supplementary I).

In the West, HR‐HPV+ OPSCC has significantly increased from 40∙5% before 2000 to 72∙2% between 2005 and 2009, indicating a time trend increase in incidence.^53^ Changes in sexual behavior and a decline in tonsillectomy rates could explain this increase in oral HPV exposure.^57^ HR‐HPV plays a crucial role in the progression of oral premalignant lesions to malignancy. HPV prevalence in oral premalignant lesions was 24∙5% (HPV16, 24∙4%), almost three times higher (OR, 3∙29) than in normal tissues.^58^ The direct evidence that HPV causes a proportion of HNC is currently lacking, predominantly due to the lack of facilities to detect active HPV infection in routine screening or diagnostic procedures, including in India. The final proof that HR‐HPV infection is causative will rest on HNC prevention following vaccination.

The average prevalence rate of HPV DNA‐positive HNC in India is 26%, based on a review of PCR‐based studies, mainly focused on OSCC (Table 3) (Supplementary I and III). Though the prevalence of HR‐HPV DNA is 33 to 58%, the E6/E7mRNA expression was observed in only 15% of these tumors. Therefore, transcriptionally active HPV‐associated OSCC in Indian patients is between 6% and 9%.^59^, ^60^ A recent study reported HPV DNA and RNA positivity in 9.4% OPSCC and 1.6% OSCC.^61^ (Table 4). Complete exome sequencing of gingivobuccal tumors revealed that 19.3% had integrated HPV sequences primarily due to HPV16.^62^ Literature focused exclusively on HR‐HPV+ OPSCC and laryngeal cancer is limited, with a single study showing a 22.8% prevalence rate.^63^ The discrepancy in the prevalence rate is primarily due to different HPV testing methods and the lack of a standard technique for HPV screening in HNC. Therefore, large‐scale epidemiological studies with a well‐established HPV detection method are required to precisely establish the role of HR‐HPV in HNC in Indian patients.

**TABLE 3:** Indian data on HPV DNA‐positive HNC.^a^ ^,^ ^b^

Tumor type	No. of studies	No. of cases	HPV prevalence weighted %	Common HR‐HPV genotypes
Head and neck cancer	16	2827	24∙5	16(73∙2%); 18(18∙5%); 31(12%); 35(4%); 56(4%)
Oral cancer	29	2387	26	16(61%); 18(27∙6%)
Oropharyngeal cancer	1	105	22∙8	16(60∙5%); 18(29∙5%); 33(4∙5%); 31(4∙5%)
Oral tongue cancer	3	277	50∙1	16(91%)
Total	49	5596	26∙4	

^a^ References are given in Supplementary III, IV.

^b^ Search strategy and selection criteria (Supplementary I).

**TABLE 4:** Indian studies on HPV DNA and RNA‐positive HNC

References	Sample type and no.	HPV DNA positivity	RNA detection method	Primers used (RNA assay)	HPV RNA positivity
Sannigrahi et al.^60^	HNC 226	29∙7% Oropharyngeal 34∙5% Oral 27%; Hypopharyngeal 16∙6%; Laryngeal 71%	RT‐PCR	HPV16E7 mRNA	9%
Gheit et al. ^61^	HNC 364	13∙7% Oropharyngeal 18∙9% Oral 11∙9% Laryngeal 16∙9%	RT‐PCR (QuantiTect Virus Kit)	Type‐specific E6 mRNA assay (20 types)	2∙7% Oropharyngeal 9∙4% Oral 1∙6% Laryngeal 1∙7%
Palve et al. ^59^	OSCC 153	58%	qRT‐PCR	HPV 16/18 E6/E7 primers	15%

## 7 PREDICTORS OF ORAL HPV INFECTION

HPV is sexually acquired, and early sexual debut, a high number of sexual partners, including oral sex partners, and previous genital warts, provide an increased risk for HPV‐positive OPSCC.^64^ Males have a three times greater risk of acquiring oral HPV infection than females.^65^ HPV may be transmitted more often from women to men. Hormonal factors play a crucial role in conferring protection against cancer in females. The estrogen and progesterone‐related factors in females are inversely correlated with the risk of HNC. Sex‐driven dimorphism in the immune system favors women, thus potentially allowing a rapid clearance of HPV.^64^ The condemned mucosa syndrome induced by alcohol, tobacco, and related carcinogens in men is aggravated by HR‐HPV infection.^66^ The male sex hormones may be a cofactor associated with oral HPV persistence, as androgens are involved in developing the larynx in adolescent boys. However, the relationship between sex steroid hormones and HNC requires further evaluation.^67^

Oral HPV prevalence remains either stable or increases significantly with age. Bimodal peaks of HPV infection at 30 and 55 years for men and about 25 and 55 years for women might be due to increased sexual activities in the young and a decrease in immunity with age.^68^ The oropharyngeal region is more susceptible to HR‐HPV infection than the oral cavity due to the reticulated epithelium in tonsillar crypts and abundant lymphoid tissues in the palatine tonsils and base of the tongue. The highly invaginated tonsillar crypts and the biofilm hosts bacterial infections and foreign materials, driving the overexpression of programmed cell death‐1 ligand‐1 (PD‐L1), which favors persistent HPV infection allowing tumorigenesis.^69^

Homosexual males (12.2%) had a higher oral HPV prevalence compared with heterosexual males (4.7%) and females (2.9%).^70^ Acquisition of oral HPV infection also occurs by salivary transmission through deep kissing.^71^ HIV patients have a threefold higher incidence of HR‐HPV positivity and an increased risk for cervical and oral cancers.^72^ The similar sexual transmission mode and HIV‐induced immune suppression may increase the risk of new HPV infection and its persistence or reactivation of latent infection.^73^ Oral HPV infection is higher in women with cervical HPV infection (20.4%) and their partners (30.7%) suggesting that genital HPV infection increases the risk of acquiring subclinical oral HPV infection.^74^ Concurrent cervical and oral HPV infection could be detected transiently in women because of increased susceptibility to HPV infection due to hereditary/immunological disturbances or represents a high‐risk group due to sexual behavior, smoking, local immunodeficiency etc.^75^ However, concurrent HPV infection dynamics must be explored to understand their clinical significance.

## 8 RISK FACTORS FOR HPV‐ASSOCIATED HNC

HPV‐associated HNC risk is highest for the tonsil (OR: 15.1), intermediate for the oropharynx (excluding tonsil) (OR: 4.3), and lowest for the oral and larynx (OR: 2.0).^76^ The relative risk of HPV‐associated OPSCC is higher in individuals with an increased lifetime number of sexual partners, increased oral‐sexual partners, oral HPV16 infection, and HPV‐16 L1 capsid protein seropositivity.^77^ Tobacco and alcohol act synergistically with HR‐HPV and have an additive effect on carcinogenesis. A review of Indian data has shown that the use of tobacco/alcohol in HPV‐related HNCs varied from 3.23% to 85.2%, with a mean prevalence of chewing tobacco (53∙34%), smoking tobacco (47∙54%), chewing and smoking tobacco (47∙05%), and alcohol use (31∙48%).^78^ Tobacco‐associated carcinogens induce genetic alterations leading to molecular changes and thus making the individual susceptible to HR‐HPV infection and its persistence. Alcohol modifies the innate immune response dose‐dependently, producing an altered inflammatory response to HPV infection.^79^ Further investigations are warranted to elucidate the association of tobacco and alcohol with HR‐HPV infection and their combined effect on oral carcinogenesis.

The relative risk of developing OSCC or OPSCC among women with cervical cancer is more significant, with a standardized incidence rate of 1.5 and 2.7, respectively.^80^ Husbands of patients with cervical cancer have a two to threefold increased risk of HPV+ OSCC. Likewise, wives of HPV+ HNC patients are at increased risk of cervical precancerous lesions.^81^ The National Cancer Registry Program, India, has reported OSCC as the most common cancer in men, while breast and cervical cancer is predominant in women.^82^ As HR‐HPV is involved in cervical and oral cancer, it becomes prudent to look for persistent HR‐HPV infection in these sites for effective prevention. Chronic inflammation or infection by specific microorganisms due to poor oral hygiene increases the cancer risk independently or by facilitating HPV acquisition and persistence.^67^ The duration between initial oral HR‐HPV infection and the onset of HPV‐related OPSCC may reach up to 20 years—thus, cofactors may play a role in carcinogenesis.^42^ These confounding factors make defining HPV as the sole causative factor for HNC challenging.

## 9 CURRENT TREATMENT PARADIGM AND PROGNOSIS OF HR‐HPV‐POSITIVE OPSCC

HPV‐related OPSCC has shown better outcomes with conventional treatment in more developed countries, with a 70% relative reduction in risk of death.^83^ Consequently, a new staging algorithm for OPSCC was recommended in the 8th AJCC/UICC guidelines (2017), where advanced‐stage tumors are now re‐categorized into a lower stage.^84^ HPV status is a recommended biomarker for patient stratification toward de‐escalation treatment regimens to minimize treatment‐associated toxicity. However, randomized clinical trials have not confirmed this.^85^, ^86^ Trials (NRG Oncology RTOG 1016) that have examined substituting cetuximab for cisplatin chemotherapy have not shown an advantage for the former.^85^ Similarly, reduced radiation doses have been ineffective when administered with chemotherapy.^86^ A recently concluded phase II randomized trial (NRG HN002) comparing reduced radiation dose with or without concurrent weekly cisplatin found an almost similar survival rate but less severe toxicity.^87^ Another randomized trial (ECOG 3311) reported favorable functional and disease outcomes after testing reduced postoperative radiotherapy following transoral robotic surgery.^88^ The current lack of evidence for de‐intensified treatment of HR‐HPV+ OPSCC reflects a selection issue, wherein most trials have identified patients solely based on p16 positivity. Ideally, the randomized trial for evaluation of treatment de‐intensification should include a well‐defined low‐risk group of HPV+ OPSCC patients (p16+ SCC, < 10 pack‐year smoking history, AJCC 8th clinical stages T1‐3N1M0) with a transcriptionally active HPV infection in the tumor. The de‐escalation strategies under investigation include immunotherapy and novel biomarkers for patient selection for de‐escalation.^89^ Currently, there is no change in the standard of care for patients with HR‐HPV+ OPSCC, and treatment remains chemoradiotherapy or surgery. The ongoing phase III studies are expected to establish new standard therapy for patients with HPV+ OPSCC (NCT02984410; NCT03811015; and NCT03452137).

The improved prognosis of patients with HPV‐associated HNC is mainly due to tumor radiosensitivity, active immune response to viral antigens, and the absence of field cancerization especially in nonsmokers. Besides, the lack of p53 mutations enables an intact apoptotic response to radiation and chemotherapy.^84^ However, patients with HR‐HPV+ tumors associated with smoking, advanced nodal stage, epidermal growth factor receptor (EGFR) overexpression, and chromosomal instability had a poor prognosis.^90^ Furthermore, patients with HR‐HPV+ HNC and p16 overexpression had better 5‐year overall survival than those with HR‐HPV+/p16− or HR‐HPV−/p16+ tumors.^84^ OPSCC patients with transcriptionally active HPV E6/E7 mRNA‐positive tumors and HPV16 E6/E7 seropositivity showed a more favorable prognosis.^91^, ^92^ A study from India has reported poorer outcomes for tobacco users with HR‐HPV+ HNC patients than nonusers.^38^ Integration‐negative tumors had better survival rates and were characterized by upregulated immune‐related genes, including CD4+, CD3+, regulatory, CD8+ T cells, NK cells, and B cells; while HPV‐integrated tumors had upregulated genes enriched for keratinization, RNA metabolism, and translation with a poor outcome similar to HPV‐negative tumors.^93^

Gene expression data reveal a subset of HPV+ OPSCC (high‐risk group) with a differential expression of 38 HPV‐correlated genes associated with poor outcomes. Among them, lower expression of IKAROS family zinc finger 3 [*IKZF3*], *ARHGAP26,* and calcium voltage‐gated channel subunit α1 D [*CACNA1D*] genes are associated with poor prognosis, representing a promising prognostic biomarker. These genes are linked to the differential HPV biology among HPV+ OPSCC.^94^ The expression levels of HPV E6/E7 direct target genes, including *RBL1*, *RBL2*, *UBE3A*, *EP300*, and *CREBBP*, in the high‐risk group of HPV+ OPSCC patients are similar to those in HPV‐negative OPSCC cases. The expression of the E1^E4 splicing isoform is significantly higher among tumors with a better outcome. E1^E4 protein inhibits E6/E7 activity, prevents E2 degradation, and downregulates mitochondrial genes.^94^ Increased full‐length E6 (fl‐E6) oncoprotein is associated with better prognosis, as it reduces mitochondrial mass and depletes antioxidant capacity by repressing the peroxisome proliferator–activated receptor gamma co‐activator 1α/estrogen‐related receptor α (PGC‐1α/ERRα) pathway.^95^ Nuclear factor erythroid 2‐related factor 2 (NRF2), a driver of antioxidant gene transcription with downstream and upstream relationships to PGC‐1α, confers poor prognosis in HPV+ cancers.^96^ HPV+ OPSCC is less hypoxic, showing a favorable treatment response. Image‐based quantification of hypoxia as a source of radioresistance is in clinical trials to guide therapy de‐escalation for HPV+ OPSCC (NCT00606294). fl‐E6 levels, along with NRF2 regulation, tumor hypoxia, and other features linked to antioxidant capacity, may aid in developing multimarker predictors of therapy response for HPV+ OPSCC. The prognostic biomarkers for HPV+ OPSCC in Indian patients are yet to be studied. Identifying prognostic biomarkers in HPV‐associated HNC is essential to decrease treatment morbidity without affecting the cure rate.

## 10 ORAL HPV PREVENTION STRATEGIES

The increasing incidence of HR‐HPV‐associated OPSCC warrants the identification of healthy individuals with a persistent oral HR‐HPV infection, as it helps to detect individuals at risk for OPSCC. HR‐HPV detection is simple as the method is noninvasive, cost‐effective, and serves as a biomarker for HPV+ HNC.^80^ However, there is no approved algorithm for the follow‐up of oral HPV16‐positive individuals compared with cervical HPV‐positive women. Moreover, the latent period between HR‐HPV infection and oncogenesis is long. The precursor lesions (premalignant or dysplastic lesions) for HR‐HPV+ OPSCC are also challenging to detect early in high‐risk patients. HPV biomarkers help to predict malignancy and disease outcomes. Detection of HPV E6 antibodies has been associated with an increased risk of developing OPSCC and was found more than 10 years before the diagnosis of HPV‐driven OPSCC. E6 serology is also considered for HPV+ OPSCC monitoring and early identification of residual disease or recurrence.^33^ Post‐treatment HPV16 E6 antibody levels remained almost similar to baseline and were not associated with the risk of recurrence. However, pretreatment HPV16 E6 seropositivity was associated with a reduced local/regional failure risk. Therefore, HPV16 E6 serology has potential clinical utility for early diagnosis of HPV+ OPSCC but is not a recommended biomarker for post‐treatment monitoring and early identification of relapses.^97^ A panel of miRs (miR‐9, miR‐106b, and miR‐93) specific to HR‐HPV+ OPSCC could serve as early biomarkers for detecting malignancy.^98^ Further studies are required to validate the significance of these biomarkers as a screening tool for OPSCC.

Another effective prevention method is HPV vaccination, but its exact role in preventing HNC is still unclear. Cervarix, a bivalent vaccine against HPV16/18; Gardasil, a quadrivalent vaccine against HPV6/11/16/18; and a recent nonavalent vaccine against 9 HPV types (Gardasil 9) are available worldwide. The United States Advisory Committee on Immunization Practices (ACIP) recommends HPV vaccination for females and males aged from 9 to 26.^99^ These vaccines have induced neutralizing antibodies in more than 98% of the vaccinated group within 1 month, offering protection for at least 10 years.^100^ Specific trial has not been conducted to check the efficacy of the HPV vaccine in preventing OPSCC. A randomized trial involving 7466 Costa Rican women receiving the bivalent HPV 16/18 vaccine has shown 93% efficiency against oral HPV16/18 infection 4 years after vaccination.^101^ A meta‐analysis has reported a 46% lower risk of developing oral HPV infection and an 80% reduction in HPV16 infection in vaccinated individuals.^102^ Data from the HIM study show that preventing persistent oropharyngeal HPV infection is likely to prevent HPV‐related HNC.^103^ A randomized phase III study (NCT04199689) is underway to evaluate the safety and efficacy of Gardasil 9 vaccination to prevent persistent oral HR‐HPV infection, a surrogate endpoint for HPV‐related HNCs.^104^ Therefore, the FDA has approved using the HPV recombinant 9‐valent vaccine, Gardasil 9, for an extended indication to prevent OPSCC. Although HPV vaccination was introduced for public health use in India in 2008, the demonstration projects had to be suspended in 2010 due to public concern following a few unrelated deaths.^105^ An observational cohort study of the vaccinated girls who received three, two, or single doses has shown a robust and sustained immune response against vaccine‐targeted HPV infection (vHPV) with similar efficacy in all the vaccinated groups at 4 years of follow‐up. The incidence and persistence of cervical vHPV infection were lower in the vaccinated group 7 years later.^106^ Due to the high incidence of OPSCC, developing and licensing an affordable gender‐neutral vaccine may benefit LMICs (low and middle‐income countries) like India. An indigenous quadrivalent HPV vaccine (Cervavac) was recently approved and will soon be introduced in the national immunization program (CTRI/2018/06/014601). This is expected to increase the coverage of HPV vaccination and subsequent elimination of HR‐HPV‐related cancers. But neither published research about this vaccine nor clinical trial data is available.

Close, long‐term monitoring of HPV types helps detect evolutionary changes.^107^ Though the oncogenic role of alpha papillomavirus in HNC is proven, the association of β‐ and γ‐HPV types such as γ11‐ and γ12‐HPV species and β1‐HPV‐5 type, previously associated with skin cancer, suggests a broader role for HPVs in HNC etiology.^108^ The therapeutic goal for HPV‐associated malignancies is HPV‐specific therapeutics, including specific molecules targeted to viral oncoproteins and therapeutic vaccines for augmenting cellular immune responses. An HPV16 E7 long‐peptide synthetic vaccine has induced a specific T‐cell immune response causing regression of lesions among women with vulvar intraepithelial neoplasia.^109^ HPV vaccination can be administered as adjuvant therapy, evidenced by the reduced risk of recurrent cervical intraepithelial neoplasia after surgical treatment.^99^ HPV vaccination for the secondary prevention of HR‐HPV‐related disease has demonstrated decreased disease recurrence and burden.^100^ However, there is no evidence regarding the role of vaccination in HR‐HPV‐related HNC, thereby warranting further research to elucidate its role in tumor response and disease outcome.

## 11 CONCLUSION

Despite an extensive literature on the role of HPV infection in HNC, most of these studies were based on identifying HPV DNA in the tumor. Nevertheless, active HPV infection is defined by detecting E6/E7 mRNA and p16 expression in tumors. Geographical variation in the contributory factors is also responsible for different HR‐HPV‐positive HNC frequencies. The prevalence of oral mucosal HPV infection in healthy individuals and their contribution to HNC remains unclear from Indian studies. Thus, it is crucial to understand the natural history of oral HPV infection in India, as this will significantly impact HPV prevention strategies in this region. The current data on oral HPV infection are based on PCR detection of HPV types that cause cervical cancer. However, sequencing studies have shown a complex oral HPV community in healthy individuals. This necessitates more research to understand the oncogenic role of other mucosal and cutaneous HPV genotypes apart from those found commonly in the cervical region. HR‐HPV‐associated OPSCC responds well to treatment with a superior survival rate. However, studies about their outcomes are limited for Indian patients and are necessary to refine current treatment regimens. Further research on HR‐HPV‐positive HNC will help understand their biology leading to more specific treatments. (Table 5).

**TABLE 5:** Potential research areas on oral HPV infection and cancer

Current status	Potential research areas
Host genetic susceptibility might lead to the persistence of both HR‐HPV and LR‐HPV	Identifying the genetic signatures could serve as potential biomarkers for identifying high‐risk individuals for HPV‐associated OPSCC.
E5 is variably expressed and associated with decreased EGFR expression in HPV+ OPSCC	The role of E5 in head and neck carcinogenesis and its correlation with EGFR should be validated.
DNA methylation of cellular or viral genes has been proposed as a second triage in cervical cancer screening programs.	The role of methylation markers for the early diagnosis of OPSCC has to be evaluated.
HPV16 E6 serology as an early biomarker for oropharyngeal cancer.	The time at which E6 seropositivity occurs during the natural history of HPV infection is still unknown.
OPSCC with HPV16 E6/E7 seropositivity showed a favorable prognosis	Their use as prognostic biomarkers has to be substantiated by larger studies
Lack of detailed evaluation of oral HPV genotypes	The role of beta and gamma HPV types in oral carcinogenesis should be explored.
Determinants and risk factors	The interaction between oral HPV infection and tobacco, alcohol, hormones, and chronic inflammation needs to be studied.
The dynamics of oral HPV carcinogenesis are mostly extrapolated from cervical cancer studies.	Large natural history studies on oral HPV infection are needed to develop oropharyngeal cancer preventive strategies.
HPV as a biomarker for de‐escalation treatment regimens.	A standard protocol has to be developed to identify patients for treatment de‐escalation trials to avoid under treatment.
Efficacy of HPV vaccination on oral HPV infection	Long‐term monitoring of oral HPV types following vaccination is recommended to check the efficacy and to detect evolutionary changes
Role of adjuvant HPV vaccination in HPV‐related HNC	HPV vaccine as adjuvant therapy in HPV‐related HNC has to be evaluated to elucidate its role in tumor response and disease outcome.

## AUTHOR CONTRIBUTIONS

**Nandimandalam Venkata Vani:** Conceptualization (equal); data curation (equal); formal analysis (equal); funding acquisition (equal); visualization (equal); writing – original draft (lead); writing – review and editing (equal). **Rajendran Madhanagopal:** Conceptualization (supporting); data curation (equal); formal analysis (supporting); visualization (supporting); writing – original draft (supporting); writing – review and editing (supporting). **Swaminathan Rajaraman:** Conceptualization (equal); formal analysis (equal); funding acquisition (equal); supervision (equal); writing – review and editing (equal). **Trivadi Sundaram Ganesan:** Conceptualization (equal); formal analysis (equal); funding acquisition (equal); supervision (lead); visualization (lead); writing – original draft (supporting); writing – review and editing (lead).

## CONFLICT OF INTEREST STATEMENT

The authors declare no potential conflicts of interest.

## Supporting information


Data S1
