# Supplementary material for: Dynamics of oral human papillomavirus infection in healthy population and head and neck cancer
Source: Cancer Med. 2023 Feb 27;12(10):11731–45. doi: 10.1002/cam4.5686 (PMC10242867; doi:10.1002/cam4.5686)
Supplement: Supplementary file 1 — Data S1 [file CAM4-12-11731-s001.docx]

**Supplementary Material:**

1. **Supplementary I. Search strategy and selection criteria**

A PubMed search identified references with the terms HPV (‘Human Papillomavirus’, ‘alpha Papillomavirus’, ‘HPV16’, ‘HPV18’, ‘HPV16/18’); Oral Cavity (‘Oral’, ‘Tongue’, ‘Oropharynx’); Head and Neck Cancers (‘Head Neck Cancer’, ‘Head Neck Neoplasm’ ‘Oral Squamous Cell Carcinoma’, ‘Oral Cancer’, ‘Tongue Cancer’, ‘Oropharyngeal Cancer’); Dynamics (‘Natural History’, ‘Persistence’, ‘Clearance’); India. Articles were retrieved using search filters and restricted to studies in humans published in English between 1990 and 2021. Additional articles were retrieved by searching the reference lists of relevant publications and review articles. The data on the pooled prevalence of oral HPV in healthy individuals and head and neck cancer was generated by analyzing the retrieved articles.

1. **Supplementary II. Global prevalence of HPV DNA positive Head and Neck Cancer.**

| **S No** | **Author** | **Year** | **Country** | **Continent** | **Cancer Type** | **Sample size (N)** | **HPV DNA+(%)** | **HPV Genotypes** | **References** |
| --- | --- | --- | --- | --- | --- | --- | --- | --- | --- |
| 1 | Aboagye et al | 2019 | Ghana | Africa | HNC | 100 | 18% | 16(94∙4%); 16/18(5∙6%) | [1](#_ENREF_1) |
| 2 | Sekee et al | 2018 | S Africa | Africa | HNC | 112 | 6∙3% | 16 (28∙6%); 11(28∙6%); 18(14∙3%); 31(14∙3%); 45(14∙3%) | [2](#_ENREF_2) |
| 3 | Kaba et al | 2014 | Ghana | Africa | HNC | 78 | 19∙2% | 16(86∙7%); 18(13∙3%) | [3](#_ENREF_3) |
| 4 | Babiker et al | 2013 | Sudan | Africa | OSCC | 100 | 8% | 16(62∙5%); 18, 31, 33 (12∙5% each) | [4](#_ENREF_4) |
| 5 | Ndiaye et al | 2013 | Senegal | Africa | HNC | 117 | 3∙4% | 16(25%); 45(50%); 35(25%) | [5](#_ENREF_5) |
| 6 | Paquette et al | 2013 | S Africa | Africa | OPSCC | 51 | 80∙4% | 16(92%); 31(5∙4%); 33(2∙7%) | [6](#_ENREF_6) |
| 7 | Ahmed et al | 2012 | Sudan | Africa | HNC | 150 | 4% | 16 (50%); 18 (33%); 33 (17%) | [7](#_ENREF_7) |
| 8 | Ahmed et al | 2010 | Sudan | Africa | OSCC | 40 | 15% | 16 (33∙3%), 18 (66∙6%) | [8](#_ENREF_8) |
| 9 | Boy et al | 2006 | S Africa | Africa | OSCC | 59 | 11% | 18(100%) | [9](#_ENREF_9) |
| 10 | Ibrahim et al | 1998 | Sudan | Africa | OSCC | 88 | 0% |  | [10](#_ENREF_10) |
| 11 | Van Rensburg et al | 1996 | S Africa | Africa | OSCC | 66 | 1∙50% | 16(50%); 11(50%) | [11](#_ENREF_11) |
| 12 | Khasawneh et al | 2020 | Jordan | Asia | HNC | 61 | 31∙10% | 16(100%) | [12](#_ENREF_12) |
| 13 | Maroun et al | 2020 | Lebanon | Asia | OPSCC | 34 | 85∙3% | 16(89∙7%); 18, 39, 52 (10∙3%); 16/52, 16/59 (3∙4%) | [13](#_ENREF_13) |
| 14 | Komolmalai et al | 2020 | Thailand | Asia | OSCC | 172 | 8% | 18(57∙1%); 16(14∙3%); 16/18(28∙6%) | [14](#_ENREF_14) |
| 15 | Purwanto et al | 2020 | Indonesia | Asia | OSCC | 78 | 17∙9% | 16(7∙1%); 18(85∙8%); 16/18(7∙1%) | [15](#_ENREF_15) |
| 16 | Kim et al | 2020 | Korea | Asia | HNC | 466 | 34∙1% | 16(84∙3%); 35(6∙9%); 33(4∙4%); 18, 58 (1∙9%); 16/18 , 16/51/68 (1∙2%); 16/52/81, 16/34, 16/31/39/6, 58/11 (0∙6%) | [16](#_ENREF_16) |
| 17 | Alsbeih et al | 2019 | Saudi Arabia | Asia | HNC | 285 | 3∙5% | 16(90%); 33(10%) | [17](#_ENREF_17) |
| 18 | Yang et al | 2019 | China | Asia | HNC | 418 | 20∙8% | 16 (Majority), 11, 35, 58 (few) | [18](#_ENREF_18) |
| 19 | Ni et al | 2019 | China | Asia | HNC | 303 | 26% | 16 (71%); 18 (4∙5%); 11 (2∙2%); 82 (2∙2%); 11/16 (4∙5%); 16/18 (6∙6%); 6/16 (2∙2%); 16/18/33 (4∙5%); 11/16/18/33 (2∙2%) | [19](#_ENREF_19) |
| 20 | Adnan Ali | 2018 | Pakistan | Asia | OSCC | 140 | 67∙9% | 16(89∙5%); 18(2∙1%); others (6∙4%) | [20](#_ENREF_20) |
| 21 | Zil-e-Rubab | 2018 | Pakistan | Asia | OSCC | 100 | 46% | 16(8∙7%); 18(10∙9%); 16/18(50%) | [21](#_ENREF_21) |
| 22 | Phusingha et al | 2017 | Thailand | Asia | OSCC | 80 | 17∙5% | 16(92∙8%); 18(7∙2%) | [22](#_ENREF_22) |
| 23 | Qatouseh et al | 2017 | Jordan | Asia | OSCC | 108 | 14∙8% | 16(81∙3%); 16/18 (18∙7%) | [23](#_ENREF_23) |
| 24 | Hosseini et al | 2017 | Iran | Asia | LC | 50 | 28% | 16(57%); 18 (42∙8%); 16/18(14∙2%) | [24](#_ENREF_24) |
| 25 | Wang et al | 2016 | China | Asia | OPSCC | 188 | 11∙7% | 16(100%) | [25](#_ENREF_25) |
| 26 | Chen et al | 2016 | China | Asia | OSCC | 40 | 0% | --- | [26](#_ENREF_26) |
| 27 | Lam et al | 2016 | China | Asia | OPSCC | 207 | 21∙7% | 16(97∙6%); 18(2∙4%) | [27](#_ENREF_27) |
| 28 | Deng et al | 2014 | Japan | Asia | HNC | 150 | 31∙3% | 16(83%); 33(8∙5%); 35(2%); 58(4∙2%); 56(2%) | [28](#_ENREF_28) |
| 29 | Hama et al | 2014 | Japan | Asia | OPSCC | 157 | 50∙3% | 16(88∙6%); 18(3∙8%); 58(2∙5%); 31, 33, 35, 52(1∙3% each) | [29](#_ENREF_29) |
| 30 | Xu et al | 2004 | China | Asia | HNC | 256 | 3∙5% | 16(77∙7%); 33(11∙1%); 16/11(11∙1%) | [30](#_ENREF_30) |
| 31 | Kim et al | 2014 | Korea | Asia | OPSCC | 74 | 28% | 16(76%); 18(29%); 33,35(4∙7% each) | [31](#_ENREF_31) |
| 32 | Maruyama et al | 2014 | Japan | Asia | HNC | 493 | 13∙8% | 16(91%); 18, 33, 58(1∙5% each); 35(4∙4%) | [32](#_ENREF_32) |
| 33 | Kawakami et al | 2014 | Japan | Asia | OPSCC | 104 | 38% | 16(92∙5%); 18(5%); 16/18(2∙5%) | [33](#_ENREF_33) |
| 34 | Nomura et al | 2014 | Japan | Asia | OPSCC | 77 | 36∙8% | 16(81∙3%); 18(6∙3%); 11, 33,35, 69(3∙1% each) | [34](#_ENREF_34) |
| 35 | Akhter et al | 2013 | Bangladesh | Asia | OSCC | 34 | 3% | -- | [35](#_ENREF_35) |
| 36 | Tural et al | 2013 | Turkey | Asia | OPSCC | 81 | 52% | 16(86%); 18(12%); 33(2%) | [36](#_ENREF_36) |
| 37 | Mizumachi et al | 2013 | Japan | Asia | OPSCC | 71 | 32% | 16(87%); 18(8∙7%); 58(4∙3%) | [37](#_ENREF_37) |
| 38 | Kabeya et al | 2012 | Japan | Asia | Tongue ca | 32 | 0% | --- | [38](#_ENREF_38) |
| 39 | Lee et al | 2012 | Taiwan | Asia | OSCC | 173 | 21% | 16(36∙6%); 18(32∙4%); 52(8∙4%) | [39](#_ENREF_39) |
| 40 | Wei et al | 2012 | China | Asia | HNC | 93 | 17% | 16(50%) ; 18 (43∙7%) ; 52 (6∙3%) | [40](#_ENREF_40) |
| 41 | Deng et al | 2011 | Japan | Asia | HNC | 150 | 30% | 16(86∙7%); 33(6∙7%); 58(4∙4%); 67(2∙2%) | [41](#_ENREF_41) |
| 42 | Saini et al | 2011 | Malayasia | Asia | OSCC | 105 | 51∙4% | HR 74∙1% (16,26, 31, 33,35,45,51,58) ; LR 25∙9% (6,11,53,54) | [42](#_ENREF_42) |
| 43 | Ishibashi et al | 2011 | Japan | Asia | OSCC | 50 | 12% | 16(16∙6%); 31(50%); 58(16∙6%); Multiple (16∙6%); others (16∙6%) | [43](#_ENREF_43) |
| 44 | Al swiahb et al | 2010 | Taiwan | Asia | OPSCC | 274 | 16∙4% | 16(77∙8%); 18(8∙9%); 16/18(6∙7%); 31, 16/31, 6/11(2∙2% each) | [44](#_ENREF_44) |
| 45 | Liu et al | 2010 | China | Asia | LC | 84 | 27∙4% | 16(100%) | [45](#_ENREF_45) |
| 46 | Lee et al | 2010 | Korea | Asia | Tongue ca | 36 | 36% | 16(85%) | [46](#_ENREF_46) |
| 47 | Zhao et al | 2009 | China | Asia | OSCC | 52 | 40∙4% | 16(63∙5%); 18(30∙8%), 11(1∙8%), 6(3∙9%) | [47](#_ENREF_47) |
| 48 | Khovidhunkit et al | 2008 | Thailand | Asia | OSCC | 32 | 1∙54% | --- | [48](#_ENREF_48) |
| 49 | Bhawal et al | 2008 | Japan | Asia | OSCC | 29 | 38% | 16(100%) | [49](#_ENREF_49) |
| 50 | Kuo et al | 2008 | Taiwan | Asia | Tonsil Ca | 92 | 75% | 16(84%); 58 (4%); 18, 33, 69(3%); 35, 66(1∙5% each) | [50](#_ENREF_50) |
| 51 | Koyama et al | 2007 | Japan | Asia | OSCC | 20 | 100% | 16(40%); 18(65%); 22(60%); 38(65%); 70(75%) | [51](#_ENREF_51) |
| 52 | Kim et al | 2007 | S korea | Asia | Tonsil Ca | 52 | 73∙1% | 16(82∙7%); 18, 33, 35, 58 (2∙6% each) | [52](#_ENREF_52) |
| 53 | Li et al | 2007 | Hong Kong | Asia | Tonsil Ca | 31 | 29% | 16(100%) | [53](#_ENREF_53) |
| 54 | Lim et al | 2007 | Malayasia | Asia | OSCC | 20 | 100% | 16(30%); 18(75%); 16/18(20%) | [54](#_ENREF_54) |
| 55 | Luo et al | 2007 | Taiwan | Asia | OSCC | 51 | 25% | HR (21∙5%); LR (4%) | [55](#_ENREF_55) |
| 56 | Sugiyama et al | 2007 | Japan | Asia | OSCC | 66 | 36% | 16(100%) | [56](#_ENREF_56) |
| 57 | Yang et al | 2004 | Taiwan | Asia | OSCC | 37 | 10∙8% | 16(50%); 18 (25%); 16/18(25%) | [57](#_ENREF_57) |
| 58 | Zhang et al | 2004 | China | Asia | OSCC | 73 | 74% | 16(79∙6%);18(33∙3%); 16/18(13%) | [58](#_ENREF_58) |
| 59 | Oh et al | 2004 | S korea | Asia | Tonsil Ca | 39 | 64% | 16(92%); 33 (4%); 6/58(4%) | [59](#_ENREF_59) |
| 60 | Jayasooriya et al | 2003 | Srilanka | Asia | OSCC | 102 | 37∙2% | 16(47∙4%); 18, 45 (8% each); 66(2∙6%); 6(8%); 11(5∙3%); Unknown(21%) | [60](#_ENREF_60) |
| 61 | Sugiyama et al | 2003 | Japan | Asia | OSCC | 86 | 34∙8% | 16(93∙3%); 18(6∙7%) | [61](#_ENREF_61) |
| 62 | Chang et al | 2003 | Taiwan | Asia | OSCC | 103 | 49∙5% | 16(57%); 18 (53%); 33(2%); (6, 11, 32, 44, 53, 68, CP8304, MM8; 8/51 [16%] | [62](#_ENREF_62) |
| 63 | Kojima et al | 2002 | Japan | Asia | OSCC | 53 | 66% | 38(100%) | [63](#_ENREF_63) |
| 64 | Chen et al | 2002 | Taiwan | Asia | OSCC | 29 | 82∙7% | 16 (85∙7%); 18(71∙4%); 6 (11∙1%); 11(3∙7%) | [64](#_ENREF_64) |
| 65 | Shin et al | 2002 | S korea | Asia | OSCC | 76 | 14∙5% | 16(36∙4%); 18(72∙7%); 33(18∙2%); 16/18 (9%); 16/18/33(9%) | [65](#_ENREF_65) |
| 66 | Niv et al | 2000 | Israel | Asia | HNC | 23 | 17∙3% | 16(100%) | [66](#_ENREF_66) |
| 67 | Cao et al | 2000 | China | Asia | OSCC | 40 | 72∙5% | 16 (52∙5%), 18(27∙5%) | [67](#_ENREF_67) |
| 68 | Tsuhako et al | 2000 | Japan | Asia | OSCC | 102 | 56∙9% | 16(24%); 18(22∙4%); 16/18(19%); 6/18(8∙6%); 6/16(12%); 11/16(1∙7%); 6/16/18(7%); 11(1∙7%) | [68](#_ENREF_68) |
| 69 | Patima Cao et al | 2000 | China | Asia | OSCC | 73 | 74% | 16, 18 | [69](#_ENREF_69) |
| 70 | Shima et al | 2000 | Japan | Asia | OSCC | 46 | 74% | 16(26∙4%); 18(73∙6%) | [70](#_ENREF_70) |
| 71 | Nishioka et al | 1999 | Japan | Asia | HNC | 74 | 16∙2% | 16(92%); 16/18(8%) | [71](#_ENREF_71) |
| 72 | Koh et al | 1998 | Korea | Asia | OSCC | 42 | 52% | 16(68%); 18(27∙3%); 33(18∙2%); 16/33(9%); 16/18(4∙5%) | [72](#_ENREF_72) |
| 73 | Ma et al | 1998 | China | Asia | LC | 102 | 58∙8% | 16(36∙7%); 18(16∙7%); 6(8∙3%); 11(3∙3%); 33(1∙7%); 6/16(13∙3%); 6/18(20%) | [73](#_ENREF_73) |
| 74 | Mineta et al | 1998 | Japan | Asia | HNC | 98 | 23∙4% | 16(85%); 18(15%) | [74](#_ENREF_74) |
| 75 | Mirza et al | 1998 | Pakistan | Asia | OSCC | 130 | 17∙7% | 16(56∙5%); 18(43∙5%) | [75](#_ENREF_75) |
| 76 | Wen et al | 1997 | China | Asia | OSCC | 45 | 31∙1% | 16(20%); 18(24∙4%); 16/18(13∙3%) | [76](#_ENREF_76) |
| 77 | Chiba et al | 1996 | Japan | Asia | OSCC | 38 | 21% | 16(100%) | [77](#_ENREF_77) |
| 78 | Lei et al | 1996 | China | Asia | OSCC | 23 | 47∙8% | 16(54∙5%); 18(27∙3%); 16/18(18∙2%) | [78](#_ENREF_78) |
| 79 | Shindoh et al | 1995 | Japan | Asia | OSCC | 77 | 31∙2% | 16(95∙8%); 16/18(4∙2%) | [79](#_ENREF_79) |
| 80 | Shidara et al | 1994 | Japan | Asia | LC | 45 | 24% | 16(82%); 18(18%) | [80](#_ENREF_80) |
| 81 | Anwar et al | 1993 | Japan | Asia | LC | 25 | 64% | 16(18∙7%); 18(87∙5%); 33(6∙2%) | [81](#_ENREF_81) |
| 82 | Ogura et al | 1991 | Japan | Asia | OPSCC | 11 | 18∙2% | 16(50%); 18(50%) | [82](#_ENREF_82) |
| 83 | Emmett et al | 2017 | Australia | Australia | OSCC | 63 | 8% | 16(60%); 18(40%) | [83](#_ENREF_83) |
| 84 | Hong et al | 2016 | Australia | Australia | OPSCC | 515 | 42∙7% | --- | [84](#_ENREF_84) |
| 85 | Antonsson et al | 2015 | Australia | Australia | HNC | 248 | 20% | 16(84%); 18(10%); 33(4%); 69(2%) | [85](#_ENREF_85) |
| 86 | Liu et al | 2014 | Australia | Australia | OPSCC | 105 | 46% | --- | [86](#_ENREF_86) |
| 87 | Hong et al | 2013 | Australia | Australia | OPSCC | 647 | 57% | 16(95%); 18(2∙4%); 16/18, 16/35(6%);35, 18/53, 51/53 (0∙5% each) | [87](#_ENREF_87) |
| 88 | Hong et al | 2010 | Australia | Australia | OPSCC | 302 | 36% | 16(85%); 18(3%); 35(1∙8%); 33,39,56, 82, 16/18(0∙9%) | [88](#_ENREF_88) |
| 89 | Mena et al | 2020 | Spain | Europe | OPSCC | 864 | 11∙7% | 16(82∙2%); 33(5∙9%); 35(4%); 18(2%); 31, 51, 58 (1% each) | [89](#_ENREF_89) |
| 90 | Tsimplaki et al | 2017 | Greece | Europe | HNC | 172 | 12∙8% | 16(50%); 6, 18, 51 (13∙6%); 33(9%) | [90](#_ENREF_90) |
| 91 | Wagner et al | 2017 | Germany | Europe | OPSCC | 359 | 20∙6% | 16(95∙9%); 18, 26, 33 (1∙4%) | [91](#_ENREF_91) |
| 92 | Faust et al | 2016 | Sweden | Europe | HNC | 275 | 39% | 16(73%); 18(4∙7%); 33(7∙5%); 6, 35(2∙8% each); 45(1∙8%); 13,31,68,73, 11/45, 16/51,16/86, 43/73, 45/52, 45/11 (0∙9% each) | [92](#_ENREF_92) |
| 93 | Schache et al | 2016 | United Kingdom | Europe | OPSCC | 1474 | 51∙8% | 16(89∙5%); 18(1∙4%); 16/33(1∙2%); 33(2∙6%); 16/18(2∙5%) | [93](#_ENREF_93) |
| 94 | Fonmarty et al | 2015 | France | Europe | OPSCC | 71 | 31% | 16(91%); 33(9%); 6(4∙5%) | [94](#_ENREF_94) |
| 95 | Dona et al | 2015 | Italy | Europe | OPSCC | 140 | 40∙7% | 16(87∙7%); 33,35(3∙6% each); 18,51,58 (1∙7% each) | [95](#_ENREF_95) |
| 96 | Henneman et al | 2015 | Netherlands | Europe | OPSCC | 146 | 35% | 16(92%); 33(5∙8%); 35(2%) | [96](#_ENREF_96) |
| 97 | Quabius et al | 2014 | Germany | Europe | HNC | 307 | 23∙5% | 16(94∙5%); 18(2∙7%); 26, 31(1∙4% each) | [97](#_ENREF_97) |
| 98 | Van Limbergen et al | 2014 | Belgium | Europe | OPSCC | 264 | 20% | -- | [98](#_ENREF_98) |
| 99 | Ljokjel et al | 2014 | Norway | Europe | OPSCC | 226 | 54∙8% | 16(96%); 18(0∙8%); 33, 35(1∙6% each) | [99](#_ENREF_99) |
| 100 | Reuschenbach et al | 2013 | Germany | Europe | OSCC | 275 | 25% | -- | [100](#_ENREF_100) |
| 101 | Kouvousi et al | 2013 | Greece | Europe | OSCC | 45 | 11∙1% | 16 (40%); 18 (20%); 6 (40%) | [101](#_ENREF_101) |
| 102 | Nasman et al | 2013 | Sweden | Europe | OPSCC | 439 | 69% | --- | [102](#_ENREF_102) |
| 103 | Nichols et al | 2013 | United Kingdom | Europe | OPSCC | 95 | 52∙6% | 16(90%); 18(4%); 67(4%); 33(2%) | [103](#_ENREF_103) |
| 104 | Evans et al | 2013 | UK | Europe | OPSCC | 83 | 55% | 16(97%), 33 (1∙5%), 18/56 (1∙5%) | [104](#_ENREF_104) |
| 105 | Laco et al | 2012 | Czech Republic | Europe | HNC | 46 | 45∙6% | 16(88∙8%); 33(11∙1%) | [105](#_ENREF_105) |
| 106 | Rautava et al | 2012 | Finland | Europe | HNC | 106 | 57% | 16(35%); 6/11 (6∙6%); 6,11(13∙7 % each)  Multiple types (45%) - 2/6/11/16/18/30/31/33/35/43/ 56/58/59/66 | [106](#_ENREF_106) |
| 107 | Kristoffersen et al | 2012 | Norway | Europe | OSCC | 50 | 16% | -- | [107](#_ENREF_107) |
| 108 | Lopes et al | 2011 | UK | Europe | OSCC | 142 | 3∙5% | 16(40%); 18(40%); 16/18(20%) | [108](#_ENREF_108) |
| 109 | St Guily et al | 2011 | France | Europe | OPSCC | 314 | 46∙5% | 16 (89∙7%); 52(3∙4%); 18, 35, 39(2∙1% each); 33(2∙7%); 31, 51, 59(0∙7%) | [109](#_ENREF_109) |
| 110 | St Guily et al | 2011 | France | Europe | OSCC | 209 | 10∙5% | 16 (95∙5%); 18(4∙5%); 39(4∙5%) | [109](#_ENREF_109) |
| 111 | Weiss et al | 2011 | Germany | Europe | OSCC | 131 | 26∙7% | 16(100%) | [110](#_ENREF_110) |
| 112 | Pannone et at | 2011 | Italy | Europe | OSCC | 38 | 11% | 16, 53, 70 (25% each); 31/44(25%) | [111](#_ENREF_111) |
| 113 | Hoffman et al | 2010 | Germany | Europe | Tonsil ca | 39 | 53∙8% | 16(90∙5%); 35(4∙8%); 16/18(4∙8%) | [112](#_ENREF_112) |
| 114 | Attner et al | 2010 | Sweden | Europe | OPSCC | 95 | 75% | 16(86%); 33(9∙8%); 35(2∙8%); 58(1∙4%) | [113](#_ENREF_113) |
| 115 | Jung et al | 2010 | France | Europe | HNC | 231 | 13% | 16(83∙3%); 33(10%); 16/33(6∙7%) | [114](#_ENREF_114) |
| 116 | Szarka et al | 2009 | Hungary | Europe | OSCC | 65 | 47∙7% | 16(58%); 11, 18(13%); 33(6∙4%); 31, 39, 51(3∙2%) | [115](#_ENREF_115) |
| 117 | Romanitan et al | 2008 | Greece | Europe | HNC | 103 | 13% | 16 (75%) | [116](#_ENREF_116) |
| 118 | Martinez et al | 2008 | Spain | Europe | OSCC | 33 | 39∙4% | 16 (84∙6%); 6(77%); 31(23%); 16/6(61∙5%); | [117](#_ENREF_117) |
| 119 | Smeets et al | 2007 | Netherlands | Europe | HNC | 48 | 30% | 16(100%) | [118](#_ENREF_118) |
| 120 | Reimers et al | 2007 | Germany | Europe | OPSCC | 106 | 28% | 16(97%); 33(3%) | [119](#_ENREF_119) |
| 121 | Campisi et al | 2006 | Italy | Europe | OSCC | 63 | 38∙1% | 18(41∙7%); 16(33∙3%); 6(16∙6%); 53(12∙5%) | [120](#_ENREF_120) |
| 122 | Nemes et al | 2006 | Hungary | Europe | OSCC | 79 | 42% | 16(82%); other HR (18%) | [121](#_ENREF_121) |
| 123 | Hammarstedt etal | 2006 | Sweden | Europe | Tonsil Ca | 203 | 49% | 16(87%); 33(3%); 35, 45 (1%); Uncharacterised (7%) | [122](#_ENREF_122) |
| 124 | Lo Muzio et al | 2005 | Italy | Europe | OSCC | 18 | 50% | 18(77∙7%); 16(22∙2%) | [123](#_ENREF_123) |
| 125 | Tachezy et al | 2005 | Czech  Republic | Europe | OPSCC | 68 | 51∙5% | 16(80%); 33 (10%); uncharacterised (10%) | [124](#_ENREF_124) |
| 126 | Wittekindt et al | 2005 | Germany | Europe | OPSCC | 34 | 53% | 16(94%); 33(6%) | [125](#_ENREF_125) |
| 127 | Hansson et al | 2005 | Sweden | Europe | HNC | 131 | 40% | 16(73%); 18(3∙8%); 33(5∙7%); 45, 58, 59, 70, 13, 32, 62, 10, 25 (2% each), 76(3∙8%) | [126](#_ENREF_126) |
| 128 | Paradiso et al | 2004 | Italy | Europe | HNC | 57 | 8∙6% | 16(60%); others (40%) | [127](#_ENREF_127) |
| 129 | Dahlgren et al | 2004 | Sweden | Europe | Tongue ca | 110 | 10∙9% | 16(75%); 33, 35 (8∙3% each); Unknown (8∙3%) | [128](#_ENREF_128) |
| 130 | Herrero et al | 2003 | Mulitcentric | Europe | OSCC | 1415 | 3∙9% | 16(94∙7%); 16/18(5∙4%); 18(3∙3%); 35(3∙3%); 33/35(3∙3%) | [129](#_ENREF_129) |
| 131 | Herrero et al | 2003 | Mulitcentric | Europe | OPSCC | 142 | 18∙3% | 16(94∙7%); 16/18(5∙4%); 18(3∙3%); 35(3∙3%); 33/35(3∙3%) | [129](#_ENREF_129) |
| 132 | Kansky et al | 2003 | Slovenia | Europe | OSCC | 62 | 8% | 16(50%), 33, 58 (25% each), unknown | [130](#_ENREF_130) |
| 133 | Ostwald et al | 2003 | Germany | Europe | OSCC | 118 | 43∙2% | 6/11 (9∙8%), 16(68∙6%); 18(31∙4%); 16/18(9∙8%) | [131](#_ENREF_131) |
| 134 | Boscolo-Rizzo et al | 2009 | Italy | Europe | UADT | 77 | 10% | 16(100%) | [132](#_ENREF_132) |
| 135 | Giovannelli et al | 2002 | Italy | Europe | OSCC | 13 | 62% | 18(87∙5%) | [133](#_ENREF_133) |
| 136 | Lindel et al | 2001 | Switzerland | Europe | OPSCC | 99 | 14% | 16(78∙5%); 33,35,45 (7% each) | [134](#_ENREF_134) |
| 137 | Mork et al | 2001 | Norway | Europe | HNC | 160 | 20% | 16(45∙5%); 6, 33(3%); 11 (6%); others (42%) | [135](#_ENREF_135) |
| 138 | Van Houten et al | 2001 | Netherlands | Europe | HNC | 84 | 23∙8% | 16(100%) | [136](#_ENREF_136) |
| 139 | Klussman et al | 2001 | Germany | Europe | HNC | 98 | 26% | 16 (84%); 33(4%); 19(8%); 5/16 and ADX1/16 (4%) | [137](#_ENREF_137) |
| 140 | Badaracco et al | 2000 | Italy | Europe | HNC | 66 | 36∙4% | 16,6 | [138](#_ENREF_138) |
| 141 | Sand et al | 2000 | Sweden | Europe | OSCC | 24 | 12∙5% | 16/6/11 (33∙3%); 18(33∙3%); Others (33∙3%) | [139](#_ENREF_139) |
| 142 | Mellin et al | 2000 | Sweden | Europe | Tonsil Ca | 60 | 43% | 16(100%); 16/33 (3∙8%) | [140](#_ENREF_140) |
| 143 | Bouda et al | 2000 | Greece | Europe | OSCC | 19 | 94∙7% | 16 (58%); 18(21%); 6(5∙3%); 16/18(5∙3%); 16/33 (5∙3%) | [141](#_ENREF_141) |
| 144 | Aggelopoulou et al | 1999 | Greece | Europe | OSCC | 81 | 49% | 16(22%); 18(44%) | [142](#_ENREF_142) |
| 145 | Andl et al | 1998 | germany | Europe | Tonsil Ca | 21 | 52∙4% | 16(72∙7%); 33 (9%); unknown (18%) | [143](#_ENREF_143) |
| 146 | Hoffman et al | 1998 | Germany | Europe | HNC | 75 | 22∙6% | 16(66∙6%); 6, 18, 45 (16∙6%) | [144](#_ENREF_144) |
| 147 | Elamin et al | 1998 | UK | Europe | OSCC | 28 | 50% | 16 (100%); 16/6 (35∙7%); 6(7%) | [145](#_ENREF_145) |
| 148 | Fouret et al | 1997 | France | Europe | HNC | 187 | 10∙7% | 16(85%); 31(10%); others (5%) | [146](#_ENREF_146) |
| 149 | Cruz et al | 1996 | Netherlands | Europe | OSCC | 35 | 54∙3% | 16(79%);Unknown (21%) | [147](#_ENREF_147) |
| 150 | Fouret et al | 1995 | France | Europe | HNC | 131 | 11∙4% | 16(73∙3%); 31(20%); other (6∙7%) | [148](#_ENREF_148) |
| 151 | Mao et al | 1995 | UK | Europe | OSCC | 26 | 30∙8% | 16(100%) | [149](#_ENREF_149) |
| 152 | Ostwald et al | 1994 | Germany | Europe | OSCC | 26 | 61∙5% | 16(43∙75%); 18(37∙5%); 6/11(12∙5%); unknown (12∙5%) | [150](#_ENREF_150) |
| 153 | Snijders et al | 1992 | Netherlands | Europe | Tonsil Ca | 10 | 100% | 16(40%); 33(30%); 16/33(10%); unknown (20%) | [151](#_ENREF_151) |
| 154 | Yeudall et al | 1991 | UK | Europe | OSCC | 39 | 46% | 16(55∙5%); 18(44∙5%) | [152](#_ENREF_152) |
| 155 | Chang et al | 1990 | Finland | Europe | OSCC | 40 | 27∙5% | 16(81∙8%); 6(9%); 18(9%) | [153](#_ENREF_153) |
| 156 | Hernandez et al | 2019 | USA | N America | OSCC | 122 | 31% | 16(71%); 52(13∙2%); 51(7∙9%); 6(7∙9%); 18(5∙2%); 39(2∙6%); 66(2∙6%) | [154](#_ENREF_154) |
| 157 | Mazul et al | 2016 | USA | N America | OPSCC | 238 | 73∙4% | 16(87∙5%); non 16 (12∙5%) | [155](#_ENREF_155) |
| 158 | Dang et al | 2015 | USA | N America | OPSCC | 100 | 33% | 16(76%) | [156](#_ENREF_156) |
| 159 | Steinau et al | 2014 | USA | N America | OPSCC | 557 | 72% | 16(60∙5%); 18(2∙5%); 33(5∙6%); 35(2%); 39(0∙9%); 31, 52 (0∙7%); 45(0∙5%); 51,56,59,58,66,68, 11,26, 69,82, 83 (2∙9%); 16/33(1∙5%); 16/18, 16/31 (0∙7%); 16/35(0∙5%); 16/45,16/52,16/54, 16/59,16/83,18/35,33/39,39/56 (0∙2%) | [157](#_ENREF_157) |
| 160 | Isayeva et al | 2014 | USA | N America | OPSCC | 102 | 63% | 16(76∙5%); 18(15∙6%); 16/18(11%) | [158](#_ENREF_158) |
| 161 | Sharma et al | 2012 | USA | N America | HNC | 294 | 24% | HR (94∙3%); LR (5∙7%) | [159](#_ENREF_159) |
| 162 | Maxwell et al | 2010 | USA | N America | OPSCC | 124 | 82% | 16(95%); 18(2%); 35(3%) | [160](#_ENREF_160) |
| 163 | Machado et al | 2010 | Canada | N America | HNC | 92 | 20∙6% | 16(63%); 6, 18, 33, 35, 45, 52/58 (5∙3% each) | [161](#_ENREF_161) |
| 164 | Lohavanichbutr et al | 2009 | USA | N America | HNC | 119 | 35% | 16(90∙6%); 32,35,45,53(2∙3% each) | [162](#_ENREF_162) |
| 165 | Pintos et al | 2008 | Canada | N America | OSCC/OP  SCC | 72 | 19∙4% | 16(92∙8%); 84(7∙2%) | [163](#_ENREF_163) |
| 166 | Liang et al | 2008 | USA | N America | Tongue ca | 51 | 2% | 16(100%) | [164](#_ENREF_164) |
| 167 | D Souza et al | 2007 | USA | N America | OPSCC | 100 | 37% | 16(62∙2%); 72(10∙8%); 62(8%); 58, 6 (5∙4% each); 18, 31, 51, 55, 61, 66, 68, 73 (2∙7% each) | [165](#_ENREF_165) |
| 168 | El - Mofty | 2006 | USA | N America | OPSCC | 20 | 60% | 16(83∙3%); 31/33 (16∙7%) | [166](#_ENREF_166) |
| 169 | Slebos et al | 2006 | USA | N America | HNC | 36 | 22∙2% | 16(100%) | [167](#_ENREF_167) |
| 170 | Ragin et al | 2006 | USA | N America | OSCC | 125 | 24% | 16(93%); 16/11, 16/33 (3∙5% each) | [168](#_ENREF_168) |
| 171 | Baez et al | 2004 | Puerto Rica | N America | HNC | 118 | 44% | 16 (100%) | [169](#_ENREF_169) |
| 172 | Smith et al | 2004 | USA | N America | HNC | 193 | 38% | 16(87%); 18(3%); 33 (10%) | [170](#_ENREF_170) |
| 173 | Ritchie et al | 2003 | USA | N America | HNC | 139 | 21% | 16(82∙75%); 18(3∙4%); 33(13∙8%) | [171](#_ENREF_171) |
| 174 | Dahlstrom et al | 2003 | USA | N America | HNC | 27 | 29∙6% | 16(100%) | [172](#_ENREF_172) |
| 175 | Ha et al | 2002 | USA | N America | HNC | 34 | 2∙9% | 16(100%) | [173](#_ENREF_173) |
| 176 | Ringstrom et al | 2002 | USA | N America | HNC | 89 | 20% | 16(100%) | [174](#_ENREF_174) |
| 177 | Schwartz et al | 2001 | USA | N America | OSCC | 254 | 24∙4% | 16(64∙5%); Others (35∙3%) | [175](#_ENREF_175) |
| 178 | Gillison et al | 2000 | USA | N America | HNC | 253 | 25% | 16(90%); 33(4∙8%); 16/31, 18, 11,unknown(1∙6%) | [176](#_ENREF_176) |
| 179 | Portugal et al | 1997 | USA | N America | HNC | 100 | 11% | 16(54∙4%); 18(9%); 16/18 (9%); 33(27∙3%) | [177](#_ENREF_177) |
| 180 | Gopalakrishnan et al | 1997 | USA | N America | OSCC | 10 | 30% | 16(100%) | [178](#_ENREF_178) |
| 181 | Pintos et al | 1999 | Canada | N America | UADT | 101 | 17% | --- | [179](#_ENREF_179) |
| 182 | Schwartz et al | 1998 | USA | N America | OSCC | 248 | 25∙8% | 16(64%); 18(3%); Others (32∙8%) | [180](#_ENREF_180) |
| 183 | Smith et al | 1998 | USA | N America | OSCC / OPSCC | 93 | 15% | 16(43%); 18, 22, 33(14∙3%); 13, 23 (7∙1%) | [181](#_ENREF_181) |
| 184 | Wilczynski et al | 1998 | USA | N America | Tonsil Ca | 22 | 64% | 16(78∙5%); 33, 59 (7%); unclassified (7%) | [182](#_ENREF_182) |
| 185 | Mao et al | 1996 | USA | N America | OSCC | 64 | 31∙3% | 16(55%); 6(15%); Unknown (10%); 6/16(10%); 16/18(10%) | [183](#_ENREF_183) |
| 186 | Anderson et al | 1994 | USA | N America | OSCC | 27 | 22% | --- | [184](#_ENREF_184) |
| 187 | Brandwein et al | 1994 | USA | N America | OSCC | 64 | 25% | 16(100%) | [185](#_ENREF_185) |
| 188 | Holladay et al | 1993 | USA | N America | OSCC | 39 | 17∙9% | 16(85∙7%); 16/18(14∙3%) | [186](#_ENREF_186) |
| 189 | Woods et al | 1993 | USA | N America | OSCC | 18 | 78% | 16(14∙3%), 18 (14∙3%), 16/18 (35∙7%); 6,/11/16/18 (28∙6%), 6/11(7∙1%) | [187](#_ENREF_187) |
| 190 | Brachman et al | 1992 | USA | N America | HNC | 30 | 10% | 16(66∙6%); 18 (33∙3%) | [188](#_ENREF_188) |
| 191 | Pakdel et al | 2021 | Brazil | N America | HNC | 108 | 23∙1% | 16(61∙5%); 18(30∙7%); 11(15∙4%) | [189](#_ENREF_189) |
| 192 | Schiavetto et al | 2021 | Brazil | S America | OPSCC | 50 | 42% | 16(100%) | [190](#_ENREF_190) |
| 193 | de Abreu | 2018 | Brazil | S America | OSCC | 90 | 3∙3% | 16(100%) | [191](#_ENREF_191) |
| 194 | Quintero et al | 2013 | Colombia | S America | HNC | 175 | 18∙9% | 16(82%); 18(18%) | [192](#_ENREF_192) |
| 195 | Kaminagakura et al | 2011 | Brazil | S America | OSCC | 114 | 19% | 16(100%); 16/18(4∙5%) | [193](#_ENREF_193) |
| 196 | Saavedra et al | 2008 | Mexico | S America | OSCC | 62 | 43∙5% | 16(55∙6%); 18(18∙5%); 33(7∙4%);35(3∙7%); 1(3∙1%); 2(3∙7%); 13(3∙7%); 32(3∙7%) | [194](#_ENREF_194) |
| 197 | Soares et al | 2008 | Brazil | S America | OSCC | 33 | 33% | 18(81∙8%); 16/18 (18∙2%) | [195](#_ENREF_195) |
| 198 | Da silva et al | 2007 | Brazil | S America | Tongue ca | 50 | 74% | Oncogenic types | [196](#_ENREF_196) |
| 199 | Rivero et al | 2006 | Brazil | S America | OSCC | 40 | 0% | --- | [197](#_ENREF_197) |
| 200 | Furrer et al | 2006 | Argentina | S America | OSCC | 14 | 42∙8% | 16(66∙6%); 16/18 (33∙3%); 11(33∙3%); | [198](#_ENREF_198) |
| 201 | Ibieta et al | 2005 | Mexico | S America | OSCC | 50 | 42% | 16(66∙6%); 33∙3% (other types) | [199](#_ENREF_199) |
| 202 | Correnti et al | 2004 | Venezuela | S America | OSCC | 16 | 50% | High risk | [200](#_ENREF_200) |
| 203 | D Percoco et al | 2001 | Venezuela | S America | OSCC | 50 | 60% | 16(73∙3%); 18(16∙6%); 16/18(10%) | [201](#_ENREF_201) |
| 204 | Miguel et al | 1998 | Brazil | S America | HNC | 45 | 11% | 16(100%) | [202](#_ENREF_202) |
| 205 | Miller et al | 1994 | Venezuela | S America | OSCC | 30 | 66∙7% | 16(69∙2%); 18(28%); 16/18(23∙8%) | [203](#_ENREF_203) |

1. **Supplementary III. Indian prevalence of HPV DNA positive Head and Neck Cancer.**

| **S No** | **Author** | **Year** | **Place** | **Cancer type** | **Sample**  **Size** | **HPV**  **DNA + (%)** | **HPV Genotypes** | **References** |
| --- | --- | --- | --- | --- | --- | --- | --- | --- |
| 1 | Vanshika et al | 2021 | Lucknow | OSCC | 108 | 13% | 16(100%) | [204](#_ENREF_204) |
| 2 | Hallikeri et al | 2019 | Karnataka | OSCC | 30 | 60% | 16 (33∙3%); 18(30%); 16/18(13∙3%) | [205](#_ENREF_205) |
| 3 | Sarmah et al | 2019 | Assam | HNC | 80 | 20% | --- | [206](#_ENREF_206) |
| 4 | Bandhary et al | 2018 | Mangalore | HNC | 88 | 2∙27% | 16(100%) | [207](#_ENREF_207) |
| 5 | Dalakoti et al | 2018 | Karnataka | OSCC | 50 | 0% | --- | [208](#_ENREF_208) |
| 6 | Grewal et al | 2018 | Punjab | OSCC | 47 | - | 16 (40∙2%); 18 (34∙04%); 16/18 (25∙53%) | [209](#_ENREF_209) |
| 7 | Chowdary et al | 2018 | Andhra Pradesh | OSCC | 20 | 55% | 16(54∙5%); 18(27∙3%); 16/18(18∙2%) | [210](#_ENREF_210) |
| 8 | Pal et al | 2018 | Kolkata | OSCC | 104 | 36∙4% | --- | [211](#_ENREF_211) |
| 9 | Palve et al | 2018 | Bangalore | OSCC | 153 | 58% | 16(91∙4%); 18(8∙6%) | [212](#_ENREF_212) |
| 10 | Pandey et al | 2018 | Varanasi | OSCC | 24 | 20∙8% | 16(100%) | [213](#_ENREF_213) |
| 11 | Kundu et al | 2018 | Assam | OSCC | 102 | 12∙6% | 16(45∙5%); 18 (27∙3%); 16/18 (27∙3%) | [214](#_ENREF_214) |
| 12 | Gheit et al | 2017 | Maharastra | HNC | 364 | 13∙7% | 16(72%); 31(12%); 18(8%); 35(4%); 56(4%) | [215](#_ENREF_215) |
| 13 | Verma et al | 2017 | Delhi | HNC | 135 | 23% | 16 (29%); 18 (16∙1%) | [216](#_ENREF_216) |
| 14 | Rajesh et al | 2017 | Karnataka | OSCC | 60 | 0% | --- | [217](#_ENREF_217) |
| 15 | Pathak et al | 2017 | Kanpur | HNC | 33 | 15∙1% | --- | [218](#_ENREF_218) |
| 16 | Bhosale et al | 2016 | Mumbai | HNC | 427 | 1∙6% | 16 (100%) | [219](#_ENREF_219) |
| 17 | Laprise C et al | 2016 | Kerala | HNC | 350 | 0% | --- | [220](#_ENREF_220) |
| 18 | Sannigrahi et al | 2016 | Chandigarh | HNC | 226 | 29∙7% | 16(47∙7%) | [221](#_ENREF_221) |
| 19 | Murthy et al | 2016 | Mumbai | HNC | 170 | 39∙4% | 16(91%); 16/18/31/33/40 (1∙5%); 16/18/31/33/40/52/53/58 (4∙5%); 39/68/73(3%); 16/31(1∙5%) | [222](#_ENREF_222) |
| 20 | Rathore et al | 2016 | Ghaziabad, UP | OSCC | 24 | 4∙20% | 16(100%) | [223](#_ENREF_223) |
| 21 | Singh et al | 2016 | Lucknow | OSCC | 43 | 7% | --- | [224](#_ENREF_224) |
| 22 | Bijina et al | 2016 | Mangalore | OSCC | 47 | 40∙4% | --- | [225](#_ENREF_225) |
| 23 | Dhanapal et al | 2015 | Chennai | OSCC | 14 | 21∙40% | 18 (100%) | [226](#_ENREF_226) |
| 24 | Parshad et al | 2015 | Rohtak | OSCC | 50 | 42% | 16 (95∙2%) / 18 (4∙7%) | [227](#_ENREF_227) |
| 25 | Singh et al | 2015 | Lucknow | OSCC | 250 | 9∙20% | 16 (30∙4%); 18(17∙4%); 16/18(26∙1%); other types (26∙1%) | [228](#_ENREF_228) |
| 26 | Krishnan et al | 2015 | Bangalore | Tongue Ca | 50 | 44% | --- | [229](#_ENREF_229) |
| 27 | Kumar R et al | 2015 | Assam | HNC | 106 | 31∙13% | 16 (81∙8%)/18 (18∙2%) | [230](#_ENREF_230) |
| 28 | Talukdar et al | 2014 | Assam | HNC | 219 | 37% | 16 (21%); 18 (73%) | [231](#_ENREF_231) |
| 29 | Patel et al | 2014 | Gujarat | OSCC | 97 | 0% | --- | [232](#_ENREF_232) |
| 30 | Ramshankar et al | 2014 | Chennai | Tongue Ca | 167 | 51% | 16(85∙18%) | [233](#_ENREF_233) |
| 31 | Chakrobarty et al | 2014 | Kolkata | OSCC | 83 | 46% | 16;18 | [234](#_ENREF_234) |
| 32 | Maitra et al | 2013 | Mumbai | OSCC | 50 | 26% | 16(22%); 18(2%); mixed (2%) | [235](#_ENREF_235) |
| 33 | Bahl et al | 2013 | Delhi | OPSCC | 105 | 22∙8% | 16(79%); 18(12%); 31(4∙5%); 33(4∙5%) | [236](#_ENREF_236) |
| 34 | Kulkarni et al | 2011 | Karnataka | OSCC | 34 | 70∙6% | 16(41∙7%), 18(54∙2%), 16/18(4∙17%) | [237](#_ENREF_237) |
| 35 | Elango et al | 2011 | Bangalore | Tongue Ca | 60 | 50% | HPV 16 (96∙6%) | [238](#_ENREF_238) |
| 36 | Barwad et al | 2011 | Chandigarh | HNC | 111 | 32∙4% | --- | [239](#_ENREF_239) |
| 37 | Chaudhary et al | 2010 | Allahabad | OSCC | 222 | 32∙4% | 16 (100%) | [240](#_ENREF_240) |
| 38 | Jalouli et al | 2010 | Allahabad | OSCC | 62 | 24% | 16 (46∙7%); 18 (20%) | [241](#_ENREF_241) |
| 39 | Bhattacharya et al | 2009 | Kolkata | HNC | 236 | 63% | 16(91∙7%); 18(3∙4%); 16/18 (4∙8%) | [242](#_ENREF_242) |
| 40 | Majumdar et al | 2009 | Kolkata | OSCC | 83 | 46% | 16/18 | [243](#_ENREF_243) |
| 41 | Ghosh et al | 2009 | Kolkata | HNC | 94 | 64% | 16 (94%); 18 (6%) | [244](#_ENREF_244) |
| 42 | Gheit et al | 2009 | Maharastra | OSCC | 65 | 27∙50% | 16 (100%) | [245](#_ENREF_245) |
| 43 | Mitra et al | 2007 | Kolkata | HNC | 86 | 69% | 16(95%); 18(5%) | [246](#_ENREF_246) |
| 44 | Mishra et al | 2006 | Delhi | OSCC | 66 | 27% | 16(100%) | [247](#_ENREF_247) |
| 45 | Koppikar et al | 2005 | Mumbai | HNC | 102 | 31% | 16(18∙75%); 18(18∙75); 8 (31∙2%); 38 (9∙4%); 11(3%); 22(3%); 80(3%); Multiple-18/18, 6/18, 38/9, 11/32, (44%) | [248](#_ENREF_248) |
| 46 | Katiyar et al | 2003 | Delhi | OSCC | 44 | 29∙5% | 16 (100%) | [249](#_ENREF_249) |
| 47 | Herrero et al | 2003 | Multicentric | OSCC | 262 | 3∙1% | 16(2∙7%); 18(0∙8%); 35 (0∙4%) | [250](#_ENREF_250) |
| 48 | Nagpal et al | 2002 | Orissa | OSCC | 110 | 33∙6% | 16(22∙7%); 18(14∙5%); 16/18 (10%) | [251](#_ENREF_251) |
| 49 | D’Costa et al | 1998 | Mumbai | OSCC | 100 | 15% | 16 (100) | [252](#_ENREF_252) |
| 50 | Balaram et al | 1995 | Kerala | OSCC | 91 | 74% | 6(13%); 11(20%); 16(42%); 18(47%); multiple inf (41%) | [253](#_ENREF_253) |

HNC – Head and Neck Cancer; OSCC – Oral squamous cell cancer; OPSCC – Oropharyngeal cancer; Tongue ca – Tongue cancer; Tonsil ca – Tonsil cancer

**IV. References (Supplementary II & III):**

1. Aboagye E, Agyemang-Yeboah F, Duduyemi BM, Obirikorang C. Human Papillomavirus Detection in Head and Neck Squamous Cell Carcinomas at a Tertiary Hospital in Sub-Saharan Africa. *Sci World J* 2019; 2561530.

2. Sekee TR, Burt FJ, Goedhals D, Goedhals J, Munsamy Y, Seedat RY. Human papillomavirus in head and neck squamous cell carcinomas in a South African cohort. *Papillomavirus Res* 2018;**6**:58–62.

3. Kaba G, Dzudzor B, Gyasi RK, et al. Human papillomavirus genotypes in a subset of head and neck squamous cell carcinoma. *West Afr J Med* 2014;**33(2)**:121–4.

4. Babiker AY, Eltom FM, Abdalaziz MS, Rahmani A, Abusail S, Ahmed HG. Screening for high-risk human papillomavirus (HR-HPV) subtypes, among Sudanese patients with oral lesions. *Int J Clin Exp Med* 2013;**6(4)**:275–81.

5. Ndiaye C, Alemany L, Diop Y, et al. The role of human papillomavirus in head and neck cancer in Senegal. *Infect Agents Cancer* 2013;**8(1)**:14.

6. Paquette C, Evans MF, Meer SS, Rajendran V, Adamson CS, Cooper K. Evidence that alpha-9 human papillomavirus infections are a major etiologic factor for oropharyngeal carcinoma in black South Africans. *Head Neck Path* 2013;**7(4)**:361–72.

7. Ahmed HG, Mustafa SA, Eltom FM, Babiker AY. Frequency and genotype of human papillomavirus among Sudanese patients with head and neck tumours. *E cancer medical science* 2012;**6**:282.

8. Ahmed HG EF. Detection of Human Papilloma Virus Types 16 and 18 among Sudanese Patients with Oral Squamous Cell Carcinoma. *Open Cancer J* 2010;**3**:130–4.

9. Boy S, Rensburg EJV, Engelbrecht S, Dreyer L, van Heerden M, van Heerden W. HPV detection in primary intra-oral squamous cell carcinomas – commensal, aetiological agent or contamination? *J Oral Path Med* 2006;**35(2)**:86–90.

10. Ibrahim SO, Bertelsen B, Kalvenes M, et al Expression of keratin 13, 14 and 19 in oral squamous cell carcinomas from Sudanese snuff dippers: Lack of association with human papillomavirus infection. *APMIS* 1998;**106**:959–69.

11. Van Rensburg EJ, Engelbrecht S, Van Heerden WF, Raubennheimer EJ, Schoub BD. Human papillomavirus DNA in oral squamous cell carcinomas from an African population sample. *Anticancer Res* 1996;**16(2)**:969–73.

12. Khasawneh AI HN, Himsawi N, Abu-Raideu J, et al. Prevalence of Human Papillomavirus Associated with Head and Neck Squamous Cell Carcinoma in Jordanian Patients. *Open Microbiol J* 2020;**14**:57–64.

13. Maroun CA, Al Feghali K, Traboulsi H, et al. HPV-related oropharyngeal cancer prevalence in a middle eastern population using E6/E7 PCR. *Infect Agents Cancer* 2020;**15(1)**:1.

14. Komolmalai N, Pongsiriwet S, Lertprasertsuke N, et al. Human Papillomavirus 16 and 18 Infection in Oral Cancer in Thailand: A Multicenter Study. *Asian Pac J Cancer Prev* 2020;**21(11)**:3349–55.

15. Purwanto DJ, Soedarsono N, Reuwpassa JO, Adisasmita AC, Ramli M, Djuwita R. The prevalence of oral high-risk HPV infection in Indonesian oral squamous cell carcinoma patients. *Oral Dis* 2020;**26(1)**:72–80.

16. Kim Y, Joo YH, Kim MS, Lee YS. Prevalence of high-risk human papillomavirus and its genotype distribution in head and neck squamous cell carcinomas. *J Pathol Transl Med* 2020;**54(5)**:411–8.

17. Alsbeih G, Al-Harbi N, Bin Judia S, et al. Prevalence of Human Papillomavirus (HPV) Infection and the Association with Survival in Saudi Patients with Head and Neck Squamous Cell Carcinoma. *Cancers* 2019;**11(6)**.

18. Yang JQ, Wu M, Liu YF, et al. Correlation between HPV infection and tumor recurrence in patients with head and neck squamous cell carcinoma in Tangshan, China. *Int J Clin Exp* 2019;**12**:10689–95.

19. Ni G, Huang K, Luan Y, et al. Human papillomavirus infection among head and neck squamous cell carcinomas in southern China. *PloS One* 2019;**14(9)**:e0221045.

20. Adnan Ali SM, Awan MS, Atif S, Ali N, Mirza Y. Correlation of human papillomavirus infection and clinical parameters with five-year survival in oral squamous cell carcinoma. *J Laryngol Otol* 2018;**132(7)**:628–35.

21. Zil e R, Baig S, Zaman U, Lucky MH. Human papillomavirus16/18: Fabricator of trouble in oral squamous cell carcinoma. *Int J Infect Dis* 2018;**69**:115–9.

22. Phusingha P, Ekalaksananan T, Vatanasapt P, et al. Human papillomavirus (HPV) infection in a case-control study of oral squamous cell carcinoma and its increasing trend in northeastern Thailand. *J Med Virol* 2017;**89(6)**:1096–101.

23. Abu Qatouseh L, Sabri I, Alkhatib I, Atwa E, Arafat T. Detection of High-Risk Human Papillomavirus Genotypes 16 and 18 in Head and Neck Squamous Cell Carcinomas in Jordan. *Asian Pac J Cancer Prev* 2017;**18(5)**:1337–41.

24. Hosseini SZ, Makvandi M, Samarbafzade A, et al. Frequency of Human Papillomavirus (HPV) 16 and 18 Detection in Paraffin-Embedded Laryngeal Carcinoma Tissue. *Asian Pac J Cancer Prev* 2017;**18(4)**:889–93.

25. Wang Z, Xia RH, Ye DX, et al. Human Papillomavirus 16 Infection and TP53 Mutation: Two Distinct Pathogeneses for Oropharyngeal Squamous Cell Carcinoma in an Eastern Chinese Population. *PloS One* 2016;**11**:e0164491.

26. Chen X-J, Sun K, Jiang W-W. Absence of high-risk HPV 16 and 18 in Chinese patients with oral squamous cell carcinoma and oral potentially malignant disorders. *Virol J* 2016;**13(1)**:81.

27. Lam EWH, Chan JYW, Chan ABW, et al. Prevalence, Clinicopathological Characteristics, and Outcome of Human Papillomavirus–Associated Oropharyngeal Cancer in Southern Chinese Patients. *Cancer Epidemiol Biomark Prev* 2016;**25(1)**:165–73.

28. Deng Z, Hasegawa M, Aoki K, et al. A comprehensive evaluation of human papillomavirus positive status and p16INK4a overexpression as a prognostic biomarker in head and neck squamous cell carcinoma. *Int J Oncol* 2014;**45(1)**:67–76.

29. Hama T, Tokumaru Y, Fujii M, et al. Prevalence of Human Papillomavirus in Oropharyngeal Cancer: A Multicenter Study in Japan. *Oncology* 2014;**87(3)**:173–82.

30. Xu Y, Liu S, Yi H, Wang J, Luo Y, Yin S. Low prevalence of human papillomavirus in head and neck squamous cell carcinoma in Chinese patients. *J Med Virol* 2015;**87(2)**:281–6.

31. Kim M-J, Ki M-S, Kim K, et al. Different protein expression associated with chemotherapy response in oropharyngeal cancer according to HPV status. *BMC Cancer* 2014;**14(1)**:824.

32. Maruyama H, Yasui T, Ishikawa-Fujiwara T, et al. Human papillomavirus and p53 mutations in head and neck squamous cell carcinoma among Japanese population. *Cancer science* 2014;**105(4)**:409–17.

33. Kawakami H, Okamoto I, Terao K, et al. Human papillomavirus DNA and p16 expression in Japanese patients with oropharyngeal squamous cell carcinoma. *Cancer Med* 2013;**2(6)**:933–41.

34. Nomura F, Sugimoto T, Kitagaki K, et al. Clinical characteristics of Japanese oropharyngeal squamous cell carcinoma positive for human papillomavirus infection. *Acta Otolaryngol* 2014;**134(12)**:1265–74.

35. Akhter M, Ali L, Hassan Z, Khan I. Association of human papillomavirus infection and oral squamous cell carcinoma in Bangladesh. *J Health Popul Nutr* 2013;**31(1)**:65–9.

36. Tural D, Eliçin O, Batur Ş, et al. Human papillomavirus is independent prognostic factor on outcome of oropharyngeal squamous cell carcinoma. *Tumor Biol* 2013;**34(6)**:3363–9.

37. Mizumachi T, Kano S, Sakashita T, et al. Improved survival of Japanese patients with human papillomavirus-positive oropharyngeal squamous cell carcinoma. *Int J Clin Oncol* 2013;**18(5)**:824–8.

38. Kabeya M, Furuta R, Kawabata K, Takahashi S, Ishikawa Y. Prevalence of human papillomavirus in mobile tongue cancer with particular reference to young patients. *Cancer Sci* 2012;**103(2)**:161–8.

39. Lee LA, Huang CG, Liao CT, et al. Human papillomavirus-16 infection in advanced oral cavity cancer patients is related to an increased risk of distant metastases and poor survival. *PloS One* 2012;**7(7)**:e40767.

40. Wei W, Shi Q, Guo F, et al. The distribution of human papillomavirus in tissues from patients with head and neck squamous cell carcinoma. *Oncol Rep* 2012;**28(5)**:1750–6.

41. Deng Z, Hasegawa M, Matayoshi S, et al. Prevalence and clinical features of human papillomavirus in head and neck squamous cell carcinoma in Okinawa, southern Japan. *Eur Arch Oto-Rhino-L* 2011;**268(11)**:1625–31.

42. Saini R, Tang TH, Zain RB, et al. SigniWcant association of high-risk human papillomavirus (HPV) but not of p53 polymorphisms with oral squamous cell carcinomas in Malaysia. *J Cancer Res Clin Oncol* 2011;**137**:311–20.

43. Ishibashi M, Kishino M, Sato S, et al. The prevalence of human papillomavirus in oral premalignant lesions and squamous cell carcinoma in comparison to cervical lesions used as a positive control. *Int J Clin Oncol* 2011;**16(6)**:646–53.

44. Al-Swiahb JN, Huang C-C, Fang F-M, et al. Prognostic Impact of p16, p53, Epidermal Growth Factor Receptor, and Human Papillomavirus in Oropharyngeal Cancer in a Betel Nut–Chewing Area. *Arch Otolaryngol–Head Neck Surg* 2010;**136(5)**:502–8.

45. Liu B, Lu Z, Wang P, Basang Z, Rao X. Prevalence of high-risk human papillomavirus types (HPV-16, HPV-18) and their physical status in primary laryngeal squamous cell carcinoma. *Neoplasma* 2010;**57(6):**594–600.

46. Lee SY, Cho NH, Choi EC, et al. Relevance of human papillomavirus (HPV) infection to carcinogenesis of oral tongue cancer. *J Oral Maxillofac Surg* 2010;**39(7)**:678-83.

47. Zhao D, Xu Qg, Chen Xm, Fan Mw. Human Papillomavirus as an Independent Predictor in Oral Squamous Cell Cancer. *Int J Oral Sci* 2009;**1(3)**:119–25.

48. Khovidhunkit SO, Buajeeb W, Sanguansin S, Poomsawat S, Weerapradist W. Detection of human papillomavirus in oral squamous cell carcinoma, leukoplakia and lichen planus in Thai patients. *Asian Pac J Cancer Prev* 2008;**9(4)**:771–5.

49. Bhawal UK, Sugiyama M, Nomura Y, Kuniyasu H, Tsukinoki K. Loss of 14-3-3 Sigma Protein Expression and Presence of Human Papillomavirus Type 16 E6 in Oral Squamous Cell Carcinoma. *Arch Otolaryngol–Head Neck Surg* 2008;**134(10)**:1055–9.

50. Kuo KT, Hsiao CH, Lin CH, Kuo LT, Huang SH, Lin MC. The biomarkers of human papillomavirus infection in tonsillar squamous cell carcinoma-molecular basis and predicting favorable outcome. *Mod Pathol* 2008;**21(4)**:376–86.

51. Koyama K, Uobe K, Tanaka A. Highly sensitive detection of HPV-DNA in paraffin sections of human oral carcinomas. *J Oral Pathol Med* 2007;**36(1)**:18–24.

52. Kim S-H, Koo B-S, Kang S, et al. HPV integration begins in the tonsillar crypt and leads to the alteration of p16, EGFR and c-myc during tumor formation. *Int J Cancer* 2007;**120(7)**:1418-25.

53. Li W, Tran N, Lee SC, et al. New evidence for geographic variation in the role of human papillomavirus in tonsillar carcinogenesis. *Pathology* 2007;**39(2)**:217–22.

54. Lim KP, Hamid S, Lau S-H, Teo S-H, Cheong SC. HPV infection and the alterations of the pRB pathway in oral carcinogenesis. *Oncol Rep* 2007;**17(6)**:1321–6.

55. Luo CW, Roan CH, Liu CJ. Human papillomaviruses in oral squamous cell carcinoma and pre-cancerous lesions detected by PCR-based gene-chip array. *Int J Oral Maxillofac Surg* 2007;**36(2)**:153–8.

56. Sugiyama M, Bhawal UK, Kawamura M, et al. Human papillomavirus-16 in oral squamous cell carcinoma: Clinical correlates and 5-year survival. *Br J Oral Maxillofac Surg* 2007;**45(2)**:116–22.

57. Yang Y-Y, Koh L-W, Tsai J-H, et al. Involvement of Viral and Chemical Factors with Oral Cancer in Taiwan. *Jpn J Clin Oncol* 2004;**34(4)**:176–83.

58. Zhang ZY, Sdek P, Cao J, Chen WT. Human papillomavirus type 16 and 18 DNA in oral squamous cell carcinoma and normal mucosa. *Int J Oral Maxillofac Surg* 2004;**33(1)**:71–4.

59. Oh TJ, Kim CJ, Woo SK, et al. Development and clinical evaluation of a highly sensitive DNA microarray for detection and genotyping of human papillomaviruses.
*J Clin Microbiol* 2004;**42(7)**:3272–80.

60. Jayasooriya PR, Kurose K, Terai M, et al. Human papillomavirus in oral cancer from Sri Lanka: prevalence and relationship with clinico-pathological parameters. *Oral Med Pathol* 2003;**8**:45–50.

61. Sugiyama M, Bhawal UK, Dohmen T, Ono S, Miyauchi M, Ishikawa T. Detection of human papillomavirus-16 and HPV-18 DNA in normal, dysplastic, and malignant oral epithelium. *Oral Surg Oral Med Oral Pathol Oral Radiol Endod* 2003;**95(5)**:594–600.

62. Chang JY-F, Lin M-C, Chiang C-P. High-Risk Human Papillomaviruses May Have an Important Role in Non–Oral Habits–Associated Oral Squamous Cell Carcinomas in Taiwan. *Am J Clin Pathol* 2003;**120(6)**:909–16.

63. Kojima A, Maeda H, Sugita Y, Tanaka S, Kameyama Y. Human papillomavirus type 38 infection in oral squamous cell carcinomas. *Oral Oncol 2*002;**38(6)**:591–6.

64. Chen PC-H, Kuo C, Pan C-C, Chou M-Y. Risk of oral cancer associated with human papillomavirus infection, betel quid chewing, and cigarette smoking in Taiwan — an integrated molecular and epidemiological study of 58 cases. *J Oral Pathol Med* 2002;**31(6)**:317–22.

65. Shin KH, Park KH, Hong HJ, et al. Prevalence of microsatellite instability, inactivation of mismatch repair genes, p53 mutation, and human papillomavirus infection in Korean oral cancer patients. *Int J Oncol* 2002;**21(2)**:297–302.

66. Niv A, Sion-Vardi N, Gatot A, Nash M, Fliss DM. Identification and typing of human papillomavirus (HPV) in squamous cell carcinoma of the oral cavity and oropharynx.
*J Laryngol Otol* 2006;**114(1)**:41–6.

67. Cao J, Zhang ZY, Patima, Zhang YX, Chen WT. Human papillomavirus infection and p53 alteration in oral squamous cell carcinoma. *Chin J Dent Res* 2000;**3(3)**:44–9.

68. Tsuhako K, Nakazato I, Miyagi J, et al. Comparative study of oral squamous cell carcinoma in Okinawa, Southern Japan and Sapporo in Hokkaido, Northern Japan; with special reference to human papillomavirus and Epstein-Barr virus infection. *J Oral Pathol Med* 2000;**29(2)**:70–9.

69. Patima, Cao J, Chen WT, Zhang ZY. Detection of high risk human papillomavirus DNA in oral squamous cell carcinoma. *Shanghai Journal of Stomatology* 2000;**9(4):**212–5.

70. Shima K, Kobayashi I, Saito I, et al. Incidence of human papillomavirus 16 and 18 infection and p53 mutation in patients with oral squamous cell carcinoma in Japan. *Br J Oral Maxillofac Surg*. 2000;**38(5)**:445–50.

71. Nishioka S, Fukushima K, Nishizaki K, et al. Human Papillomavirus as a Risk Factor for Head and Neck Cancers - A Case-control Study. *Acta Otolaryngol* 1999;**119(540)**:77–80.

72. Koh JY, Cho NP, Kong G, Lee JD, Yoon K. p53 mutations and human papillomavirus DNA in oral squamous cell carcinoma: correlation with apoptosis. *Br J Cancer* 1998;**78(3)**:354–9.

73. Ma X-L, Ueno K, Pan Z-M, Hi S-Z, Ohyama M, Eizuru Y. Human papillomavirus DNA sequences and p53 over-expression in laryngeal squamous cell carcinomas in Northeast China. *J Med Virol* 1998;**54(3)**:186–91.

74. Mineta H, Ogino T, Amano HM, et al. Human papillomavirus (HPV) type 16 and 18 detected in head and neck squamous cell carcinoma. *Anticancer Res* 1998;**18(6B)**:476–5–8.

75. Mirza T. Molecular analysis of human papillomavirus and oncosuppressor genes in tobacco related oral cancer. *Pak J Otolaryngol -Head Neck Surg* 1998;**14**:27–32.

76. Wen S, Tsuji T, Li X, Mizugaki Y, Hayatsu Y, Shinozaki F. Detection and analysis of human papillomavirus 16 and 18 homologous DNA sequences in oral lesions. *Anticancer Res* 1997;**17(1A)**:307–11.

77. Chiba I, Shindoh M, Yasuda M, et al. Mutations in the p53 gene and human papillomavirus infection as significant prognostic factors in squamous cell carcinomas of the oral cavity. *Oncogene* 1996;**12(8)**:1663–8.

78. Lei L, Li H, Sun Y. Study of HPV in oral squamous cell carcinoma. *Chinese J stomatol* 1996;**31(6)**:375–7.

79. Shindoh M, Chiba I, Yasuda M, et al. Detection of human papillomavirus DNA sequences in oral squamous cell carcinomas and their relation to p53 and proliferating cell nuclear antigen expression. *Cancer* 1995;**76(9)**:1513–21.

80. Shidara K, Suzuki T, Hara F, Nakajima T. Lack of synergistic association between human papillomavirus and ras gene point mutation in laryngeal carcinomas. *Laryngoscope* 1994;**104(8)**:1008–12.

81. Anwar K, Nakakuki K, Naiki H, Inuzuka M. ras Gene mutations and HPV infection are common in human laryngeal carcinoma. *Int J Cancer* 1993;**53(1)**:22–8.

82. Ogura H, Watanabe S, Fukushima K, Masuda Y, Fujiwara T, Yoshiro Y. Presence of Human Papillomavirus Type 18 DNA in a Pharyngeal and a Laryngeal Carcinoma. *Jpn J Cancer Res* 1991;**82(11)**:1184–6.

83. Emmett S, Jenkins G, Boros S, Whiteman DC, Panizza B, Antonsson A. Low prevalence of human papillomavirus in oral cavity squamous cell carcinoma in Queensland, Australia. *ANZ J Surg* 2017;**87(9)**:714–9.

84. Hong A, Lee CS, Jones D, et al. Rising prevalence of human papillomavirus-related oropharyngeal cancer in Australia over the last 2 decades. *Head Neck* 2016;**38(5)**:743–50.

85. Antonsson A, Neale RE, Boros S, et al. Human papillomavirus status and p16(INK4A) expression in patients with mucosal squamous cell carcinoma of the head and neck in Queensland, Australia. *Cancer Epidemiol* 2015 Apr;**39(2)**:174–81.

86. Liu J, Zhang M, Rose B, et al. Ki67 Expression has Prognostic Significance in Relation to Human Papillomavirus Status in Oropharyngeal Squamous Cell Carcinoma. *Ann Surg Oncol* 2015;**22(6)**:1893–900.

87. Hong A, Jones D, Chatfield M, et al. HPV status of oropharyngeal cancer by combination HPV DNA/p16 testing: biological relevance of discordant results. *Ann Surg Oncol* 2013;**20 Suppl 3**:S450–8.

88. Hong AM, Grulich AE, Jones D, et al. Squamous cell carcinoma of the oropharynx in Australian males induced by human papillomavirus vaccine targets. *Vaccine* 2010;**28(19)**:3269–72.

89. Mena M, Frias-Gomez J, Taberna M, et al. Epidemiology of human papillomavirus-related oropharyngeal cancer in a classically low-burden region of southern Europe. *Sci Rep* 2020;**10(1)**:13219.

90. Tsimplaki E, Argyri E, Sakellaridis A, Kyrodimos E, Xesfyngi D, Panotopoulou E. Oropharyngeal and laryngeal but not oral cancers are strongly associated with high-risk human papillomavirus in 172 Greek patients. *J Med Virol* 2017;**89(1)**:170-6.

91. Wagner S, Wittekindt C, Sharma SJ, et al. Human papillomavirus association is the most important predictor for surgically treated patients with oropharyngeal cancer. *Br J Cancer* 2017;**116(12)**:1604–11.

92. Faust H, Eldenhed Alwan E, Roslin A, Wennerberg J, Forslund O. Prevalence of human papillomavirus types, viral load and physical status of HPV16 in head and neck squamous cell carcinoma from the South Swedish Health Care Region. *J Gen Virol* 2016;**97(11)**:2949–56.

93. Schache AG, Powell NG, Cuschieri KS, et al. HPV-Related Oropharynx Cancer in the United Kingdom: An Evolution in the Understanding of Disease Etiology. *Cancer Res* 2016;**76(22)**:6598–606.

94. Fonmarty D, Cherrière S, Fleury H, et al. Study of the concordance between p16 immunohistochemistry and HPV-PCR genotyping for the viral diagnosis of oropharyngeal squamous cell carcinoma. *Eur Ann Otorhinolaryngol Head Neck Dis.* 2015;**132(3)**:135–9.

95. Donà MG, Spriano G, Pichi B, et al. Human papillomavirus infection and p16 overexpression in oropharyngeal squamous cell carcinoma: a case series from 2010 to 2014.
*Future Microbiol* 2015;**10(8)**:1283–91.

96. Henneman R, Van Monsjou HS, Verhagen CV, et al. Incidence Changes of Human Papillomavirus in Oropharyngeal Squamous Cell Carcinoma and Effects on Survival in the Netherlands Cancer Institute, 1980-2009. *Anticancer Res* 2015;**35(7)**:4015–22.

97. Quabius ES, Haag J, Kühnel A, et al. Geographical and anatomical influences on human papillomavirus prevalence diversity in head and neck squamous cell carcinoma in Germany. *Int J Oncol* 2015;**46(1)**:414–22.

98. Van Limbergen EJ, Dok R, Laenen A, et al. HPV-related oropharyngeal cancers in Flanders (Belgium): a multicenter study. *B-Ent* 2014;**10(1)**:7–14.

99. Ljøkjel B, Lybak S, Haave H, Olofsson J, Vintermyr OK, Aarstad HJ. The impact of HPV infection on survival in a geographically defined cohort of oropharynx squamous cell carcinoma (OPSCC) patients in whom surgical treatment has been one main treatment. *Acta Oto-Laryngologica* 2014;**134(6)**:636–45.

100. Reuschenbach M, Kansy K, Garbe K, et al. Lack of evidence of human papillomavirus-induced squamous cell carcinomas of the oral cavity in southern Germany.
*Oral Oncol* 2013;**49(9)**:937–42.

101. Kouvousi M, Xesfyngi D, Tsimplaki E, et al. Prevalence of human papillomavirus in 45 greek patients with oral cancer. *J Oncol* 2013:**756510**.

102. Näsman A,Andersson E, Marklund L, et al. HLA Class I and II Expression in Oropharyngeal Squamous Cell Carcinoma in Relation to Tumor HPV Status and Clinical Outcome. *PloS One* 2013;**8**:e77025.

103. Nichols AC, Dhaliwal SS, Palma DA, et al. Does HPV type affect outcome in oropharyngeal cancer? *J Otolaryngol- Head N* 2013;**42(1)**:9.

104. Evans M, Newcombe R, Fiander A, et al. Human Papillomavirus-associated oropharyngeal cancer: an observational study of diagnosis, prevalence and prognosis in a UK population. *BMC Cancer* 2013;**13(1)**:220.

105. Laco J, Vosmikova H, Novakova V, et al. The role of high-risk human papillomavirus infection in oral and oropharyngeal squamous cell carcinoma in non-smoking and non-drinking patients: a clinicopathological and molecular study of 46 cases.
*Virchows Arch* 2011;**458(2)**:179–87.

106. Rautava J, Kuuskoski J, Syrjänen K, Grenman R, Syrjänen S. HPV genotypes and their prognostic significance in head and neck squamous cell carcinomas. *J Clin Virol* 2012;**53(2)**:116–20.

107. Kristoffersen AK, Enersen M, Kverndokk E, et al. Human papillomavirus subtypes in oral lesions compared to healthy oral mucosa. *J Clin Virol*. 2012;**53(4)**:364–6.

108. Lopes V, Murray P, Williams H, Woodman C, Watkinson J, Robinson M. Squamous cell carcinoma of the oral cavity rarely harbours oncogenic human papillomavirus. *Oral Oncol* 2011;**47(8)**:698–701.

109. St Guily JL, Jacquard A-C, Prétet J-L, et al. Human papillomavirus genotype distribution in oropharynx and oral cavity cancer in France—The EDiTH VI study. *J Clin Virol* 2011;**51(2)**:100–4.

110. Weiss D, Koopmann M, Rudack C. Prevalence and impact on clinicopathological characteristics of human papillomavirus-16 DNA in cervical lymph node metastases of head and neck squamous cell carcinoma. *Head Neck* 2011;**33(6)**:856–62.

111. Pannone G, Santoro A, Carinci F, et al. Double Demonstration of Oncogenic High Risk Human Papilloma Virus DNA and HPV-E7 Protein in Oral Cancers. *Int J Immunopathol Pharmacol* 2011;**24(2_suppl)**:95–101.

112. Hoffmann M, Ihloff AS, Görögh T, et al. p16INK4a overexpression predicts translational active human papillomavirus infection in tonsillar cancer. *Int J Cancer* 2010;**127(7)**:1595–602.

113. Attner P, Du J, Näsman A, et al. The role of human papillomavirus in the increased incidence of base of tongue cancer. *Int J Cancer* 2010;**126(12)**:2879–84.

114. Jung AC, Briolat J, Millon R, et al. Biological and clinical relevance of transcriptionally active human papillomavirus (HPV) infection in oropharynx squamous cell carcinoma. *Int J Cancer* 2010;**126(8)**:1882–94.

115. Szarka K, Tar I, Fehér E, et al. Progressive increase of human papillomavirus carriage rates in potentially malignant and malignant oral disorders with increasing malignant potential. *Oral Microbiol Immunol* 2009;**24(4)**:314–8.

116. Romanitan M, Näsman A, Ramqvist T, et al. Human Papillomavirus Frequency in Oral and Oropharyngeal Cancer in Greece. *Anticancer Res* 2008;**28(4B)**:2077–80.

117. Llamas-Martínez S, Esparza-Gómez G, Campo-Trapero J, et al. Genotypic Determination by PCR-RFLP of Human Papillomavirus in Normal Oral Mucosa, Oral Leukoplakia and Oral Squamous Cell Carcinoma Samples in Madrid (Spain). *Anticancer Res* 2008;28**(6A)**:3733–41.

118. Smeets SJ, Hesselink AT, Speel E-JM, et al. A novel algorithm for reliable detection of human papillomavirus in paraffin embedded head and neck cancer specimen. *Int J Cancer* 2007;**121(11**):2465–72.

119. Reimers N, Kasper HU, Weissenborn SJ, et al. Combined analysis of HPV-DNA, p16 and EGFR expression to predict prognosis in oropharyngeal cancer. *Int J Cancer* 2007;**120(8)**:1731–8.

120. Campisi G, Giovannelli L, Calvino F, et al. HPV infection in relation to OSCC histological grading and TNM stage. Evaluation by traditional statistics and fuzzy logic model. *Oral Oncol* 2006;**42(6)**:638–45.

121. Nemes JA, Deli L, Nemes Z, Márton IJ. Expression of p16(INK4A), p53, and Rb proteins are independent from the presence of human papillomavirus genes in oral squamous cell carcinoma.  *Oral Surg Oral Med Oral Pathol Oral Radiol* 2006;**102(3)**:344–52.

122. Hammarstedt L, Lindquist D, Dahlstrand H, et al. Human papillomavirus as a risk factor for the increase in incidence of tonsillar cancer. *Int J Cancer* 2006;**119(11)**:2620–3.

123. Muzio LL, D'Angelo M, Procaccini M, et al. Expression of cell cycle markers and human papillomavirus infection in oral squamous cell carcinoma: Use of fuzzy neural networks. *Int J Cancer* 2005;**115(5)**:717–23.

124. Tachezy R, Klozar J, Saláková M, et al. HPV and other risk factors of oral cavity/oropharyngeal cancer in the Czech Republic. *Oral Dis* 2005;**11(3)**:181–5.

125. Wittekindt C, Gültekin E, Weissenborn SJ, Dienes HP, Pfister HJ, Klussmann JP. Expression of p16 Protein Is Associated with Human Papillomavirus Status in Tonsillar Carcinomas and Has Implications on Survival. *Curr Res Head Neck Cancer* 2004;**62**:72–80

126. Hansson BG, Rosenquist K, Antonsson A, et al. Strong association between infection with human papillomavirus and oral and oropharyngeal squamous cell carcinoma: A population-based case-control study in southern Sweden. *Acta Otolaryngol* 2005;**125(12)**:1337–44.

127. Paradiso A, Ranieri G, Stea B, et al. Altered p16INK4a and Fhit expression in carcinogenesis and progression of human oral cancer. *Int J Oncol*  2004;**24(2)**:249–55.

128. Dahlgren L, Dahlstrand H, Lindquist D, et al. Human papillomavirus is more common in base of tongue than in mobile tongue cancer and is a favorable prognostic factor in base of tongue cancer patients. *Int J Cancer* 2004;**112(6)**:1015–9.

129. Herrero R, Castellsagué X, Pawlita M, et al. Human Papillomavirus and Oral Cancer: The International Agency for Research on Cancer Multicenter Study. *J Natl Cancer Inst* 2003;**95(23)**:1772–83.

130. Kansky AA, Poljak M, Seme K, et al. Human papillomavirus DNA in oral squamous cell carcinomas and normal oral mucosa. *Acta Virol* 2003;**47(1)**:11–6.

131. Ostwald C, Rutsatz K, Schweder J, Schmidt W, Gundlach K, Barten M. Human papillomavirus 6/11, 16 and 18 in oral carcinomas and benign oral lesions. *Med Microbiol Immunol* 2003;**192(3)**:145–8.

132. Boscolo-Rizzo P, Da Mosto MC, Fuson R, Frayle-Salamanca H, Trevisan R, Del Mistro A. HPV-16 E6 L83V variant in squamous cell carcinomas of the upper aerodigestive tract. *J Cancer Res Clin Oncol* 2009;**135(4)**:559–66.

133. Giovannelli L, Campisi G, Lama A, et al. Human Papillomavirus DNA in Oral Mucosal Lesions. *J Infect Dis* 2002;**185(6)**:833–6.

134. Lindel K, Beer KT, Laissue J, Greiner RH, Aebersold DM. Human papillomavirus positive squamous cell carcinoma of the oropharynx. *Cancer* 2001;**92(4)**:805–13.

135. Mork J, Lie AK, Glattre E, et al. Human Papillomavirus Infection as a Risk Factor for Squamous-Cell Carcinoma of the Head and Neck. *N Engl J Med* 2001;**344(15)**:1125–31.

136. van Houten VMM, Snijders PJF, van den Brekel MWM, et al. Biological evidence that human papillomaviruses are etiologically involved in a subgroup of head and neck squamous cell carcinomas. *Int J Cancer* 2001;**93(2)**:232–5.

137. Klussmann JP, Weissenborn SJ, Wieland U, et al. Prevalence, distribution, and viral load of human papillomavirus 16 DNA in tonsillar carcinomas. *Cancer* 2001;**92(11)**:2875–84.

138. Badaracco G, Venuti A, Morello R, Muller A, Marcante ML. Human papillomavirus in head and neck carcinomas: prevalence, physical status and relationship with clinical/pathological parameters. *Anticancer Res* 2000;**20(2B)**:1301–5.

139. Sand L, Jalouli J, Larsson PA, Hirsch JM. Human papillomaviruses in oral lesions. *Anticancer Res* 2000;**20(2B)**:1183–8.

140. Mellin H, Friesland S, Lewensohn R, Dalianis T, Munck-Wikland E. Human papillomavirus (HPV) DNA in tonsillar cancer: Clinical correlates, risk of relapse, and survival. *Int J Cancer* 2000;**89(3)**:300–4.

141. Bouda M, Gorgoulis VG, Kastrinakis NG, et al. "High risk" HPV types are frequently detected in potentially malignant and malignant oral lesions, but not in normal oral mucosa.  *Mod Pathol* 2000;**13(6)**:644–53.

142. Aggelopoulou EP, Skarlos D, Papadimitriou C, Kittas C, Troungos C. Human papillomavirus DNA detection in oral lesions in the Greek population. *Anticancer Res* 1999;**19(2B)**:1391–5.

143. Andl T, Kahn T, Pfuhl A, et al. Etiological Involvement of Oncogenic Human Papillomavirus in Tonsillar Squamous Cell Carcinomas Lacking Retinoblastoma Cell Cycle Control. *Cancer Res* 1998;**58(1)**:5–12.

144. Markus Hoffmann TKCGMTGBMLJAW. Prevalence of Human Papillomavirus in Squamous Cell Carcinoma of the Head and Neck Determined by Polymerase Chain Reaction and Southern Blot Hybridization: Proposal for Optimized Diagnostic Requirements.
*Acta Otolaryngol* 1998;**118(1)**:138–44.

145. Elamin F, Steingrimsdottir H, Wanakulasuriya S, Johnson N, Tavassoli M. Prevalence of human papillomavirus infection in premalignant and malignant lesions of the oral cavity in U.K. subjects: a novel method of detection. *Oral Oncol* 1998;**34(3)**:191–7.

146. Fouret P, Monceaux G, Temam S, Lacourreye L, St Guily JL. Human papillomavirus in head and neck squamous cell carcinomas in nonsmokers. *Arch Otolaryngol Head Neck Surg* 1997;**123(5)**:513–6.

147. Cruz IBF, Snijders PJF, Steenbergen RDM, et al. Age-dependence of human papillomavirus DNA presence in oral squamous cell carcinomas. *Eur J Cancer B Oral Oncol* 1996;**32(1)**:55–62.

148. Fouret P, Martin F, Flahault A, Saint-Guily JL. Human papillomavirus infection in the malignant and premalignant head and neck epithelium. *Am J Surg Pathol* 1995;**4(2)**:122–7.

149. Mao EJ. Prevalence of human papillomavirus 16 and nucleolar organizer region counts in oral exfoliated cells from normal and malignant epithelia.
*Oral Surg Oral Med Oral Pathol Oral Radiol Endod*. 1995;**80(3)**:320–9.

150. Ostwald C, Müller P, Barten M, et al. Human papillomavirus DMA in oral squamous cell carcinomas and normal mucosa. *J Oral Pathol Med* 1994;**23(5)**:220–5.

151. Snijders PJF, Cromme FV, Van Brule AJCD, et al. Prevalence and expression of human papillomavirus in tonsillar carcinomas, indicating a possible viral etiology. *Int J Cancer* 1992;**51(6)**:845–50.

152. Yeudall WA, Campo MS. Human Papillomavirus DNA in Biopsies of Oral Tissues. *J Gen Virol* 1991;**72(1)**:173–6.

153. Chang F, SyrjÄnen S, Nuutinen J, KÄrjÄ J, SyrjÄnen K. Detection of human papillomavirus (HPV) DNA in oral squamous cell carcinomas by in situ hybridization and polymerase chain reaction. *Arch Dermatol Res* 1990;**282(8)**:493–7.

154. Hernandez BY, Lynch CF, Chan OTM, et al. Human papillomavirus DNA detection, p16(INK4a), and oral cavity cancer in a U.S. population. *Oral oncol* 2019;**91**:92–6.

155. Mazul AL, Rodriguez-Ormaza N, Taylor JM, et al. Prognostic significance of non-HPV16 genotypes in oropharyngeal squamous cell carcinoma. *Oral oncol* 2016;**61**:98–103.

156. Dang J, Feng Q, Eaton KD, Jang H, Kiviat NB. Detection of HPV in oral rinse samples from OPSCC and non-OPSCC patients. *BMC Oral Health* 2015;**15(1)**:126.

157. Steinau M, Saraiya M, Goodman MT, et al. Human papillomavirus prevalence in oropharyngeal cancer before vaccine introduction, United States. *Emerg Infect Dis* 2014;**20(5)**:822–8.

158. Isayeva T, Xu J, Dai Q, et al. African Americans with oropharyngeal carcinoma have significantly poorer outcomes despite similar rates of human papillomavirus–mediated carcinogenesis. *Hum Pathol*. 2014;**45(2)**:310–9.

159. Sharma A, Méndez E, Yueh B, et al. Human papillomavirus-positive oral cavity and oropharyngeal cancer patients do not have better quality-of-life trajectories. *Otolaryngol Head Neck Surg* 2012;**146(5)**:739–45.

160. Maxwell JH, Kumar B, Feng FY, et al. Tobacco use in human papillomavirus-positive advanced oropharynx cancer patients related to increased risk of distant metastases and tumor recurrence. *Clin Cancer Res* 2010;**16(4)**:1226–35.

161. Machado J, Reis PP, Zhang T, et al. Low prevalence of Human Papillomavirus in oral cavity carcinomas. *Head Neck Oncol* 2010;**2(1)**:6.

162. Lohavanichbutr P, Houck J, Fan W, et al. Genomewide Gene Expression Profiles of HPV-Positive and HPV-Negative Oropharyngeal Cancer: Potential Implications for Treatment Choices. *Arch Otolaryngol–Head Neck Surg* 2009;**135(2)**:180–8.

163. Pintos J, Black MJ, Sadeghi N, et al. Human papillomavirus infection and oral cancer: A case-control study in Montreal, Canada. *Oral Oncol* 2008;**44(3)**:242–50.

164. Liang X-H, Lewis J, Foote R, Smith D, Kademani D. Prevalence and Significance of Human Papillomavirus in Oral Tongue Cancer: The Mayo Clinic Experience. *J Oral Maxillofac Surg* 2008;**66(9)**:1875–80.

165. D'Souza G, Kreimer AR, Viscidi R, et al. Case–Control Study of Human Papillomavirus and Oropharyngeal Cancer. *N Engl J Med* 2007;**356(19)**:1944–56.

166. El-Mofty SK, Patil S. Human papillomavirus (HPV)-related oropharyngeal nonkeratinizing squamous cell carcinoma: Characterization of a distinct phenotype. *Oral Surg Oral Med Oral Pathol Oral Radiol Endodont*. 2006;**101(3)**:339–45.

167. Slebos RJC, Yi Y, Ely K, et al. Gene Expression Differences Associated with Human Papillomavirus Status in Head and Neck Squamous Cell Carcinoma. *Clin Cancer Res* 2006;**12(3)**:701–9.

168. Ragin CCR, Taioli E, Weissfeld JL, et al. 11q13 amplification status and human papillomavirus in relation to p16 expression defines two distinct etiologies of head and neck tumours. *Br J Cancer* 2006;**95(10)**:1432–8.

169. Báez A, Almodóvar JI, Cantor A, et al. High frequency of HPV16-associated head and neck squamous cell carcinoma in the Puerto Rican population. *Head Neck* 2004;**26(9)**:778–84.

170. Smith EM, Ritchie JM, Summersgill KF, et al. Age, sexual behavior and human papillomavirus infection in oral cavity and oropharyngeal cancers. *Int J Cancer* 2004;**108(5)**:766–72.

171. Ritchie JM, Smith EM, Summersgill KF, et al. Human papillomavirus infection as a prognostic factor in carcinomas of the oral cavity and oropharynx. *Int J Cancer* 2003;**104(3)**:336–44.

172. Dahlstrom KR, Adler-Storthz K, Etzel CJ, et al. Human papillomavirus type 16 infection and squamous cell carcinoma of the head and neck in never-smokers: a matched pair analysis. *Clin Cancer Res* 2003;**9(7)**:2620–6.

173. Ha PK, Pai SI, Westra WH, et al. Real-time quantitative PCR demonstrates low prevalence of human papillomavirus type 16 in premalignant and malignant lesions of the oral cavity. *Clin Cancer Res* 2002;**8(5)**:1203–9.

174. Ringström E, Peters E, Hasegawa M, Posner M, Liu M, Kelsey KT. Human Papillomavirus Type 16 and Squamous Cell Carcinoma of the Head and Neck. *Clin Cancer Res* 2002;**8(10)**:3187–92.

175. Schwartz SR, Yueh B, McDougall JK, Daling JR, Schwartz SM. Human Papillomavirus Infection and Survival in Oral Squamous Cell Cancer: A Population-Based Study. *Otolaryngol–Head Neck Surg* 2001;**125(1)**:1–9.

176. Gillison ML, Koch WM, Capone RB, et al. Evidence for a Causal Association Between Human Papillomavirus and a Subset of Head and Neck Cancers. *J Natl Cancer Inst* 2000;**92(9)**:709–20.

177. Portugal LG, Goldenberg JD, Wenig BL, et al. Human Papillomavirus Expression and p53 Gene Mutations in Squamous Cell Carcinoma. *Arch Otolaryngol Head Neck Sur* 1997;**123(11)**:1230–4.

178. Gopalakrishnan R, Weghorst CM, Lehman TA, et al. Mutated and wild-type p53 expression and HPV integration in proliferative verrucous leukoplakia and oral squamous cell carcinoma. *Oral Surg Oral Med Oral Pathol Oral Radiol Endod* 1997;**83(4)**:471–7.

179. Pintos J, Franco EL, Black MJ, Bergeron J, Arella M. Human papillomavirus and prognoses of patients with cancers of the upper aerodigestive tract. *Cancer* 1999;**85(9)**:1903–9.

180. Schwartz SM, Daling JR, Doody DR, et al. Oral cancer risk in relation to sexual history and evidence of human papillomavirus infection. *J Natl Cancer Inst* 1998;**90(21)**:1626–36.

181. Smith EM, Hoffman HT, Summersgill KS, Kirchner HL, Turek LP, Haugen TH. Human papillomavirus and risk of oral cancer. *Laryngoscope* 1998;**108(7)**:1098–103.

182. Wilczynski SP, Lin BT, Xie Y, Paz IB. Detection of human papillomavirus DNA and oncoprotein overexpression are associated with distinct morphological patterns of tonsillar squamous cell carcinoma. *Am J Surg Pathol* 1998;**152(1)**:145–56.

183. Mao E-J, Schwartz SM, Daling JR, Oda D, Tickman L, Beckmann AM. Human papilloma viruses and p53 mutations in normal, pre-malignant and malignant oral epithelia. *Int J Cancer.* 1996;**69(2)**:152–8.

184. Anderson JA, Irish JC, McLachlin CM, Ngan BY. H-ras oncogene mutation and human papillomavirus infection in oral carcinomas. *Arch Otolaryngol Head Neck Surg* 1994;**120(7)**:755–60.

185. Brandwein M, Zeitlin J, Nuovo GJ, et al. HPV detection using "hot start" polymerase chain reaction in patients with oral cancer: a clinicopathological study of 64 patients. *Mod pathol* 1994;**7(7)**:720–7.

186. Holladay EB, Gerald WL. Viral Gene Detection in Oral Neoplasms Using the Polymerase Chain Reaction. *Am J Clin Pathol* 1993;**100(1)**:36–40.

187. Woods KV, Shillitoe EJ, Spitz MR, Schantz SP, Adler-Storthz K. Analysis of human papillomavirus DNA in oral squamous cell carcinomas. *J Oral Pathol Med* 1993;**22(3)**:101–8.

188. Brachman DG, Graves D, Vokes E, et al. Occurrence of <em>p53</em> Gene Deletions and Human Papilloma Virus Infection in Human Head and Neck Cancer. *Cancer Res* 1992;**52(17)**:4832–6.

189. Pakdel F, Farhadi A, Pakdel T, et al. The frequency of high-risk human papillomavirus types, HPV16 lineages, and their relationship with p16(INK4a) and NF-κB expression in head and neck squamous cell carcinomas in Southwestern Iran.
*Braz J Microbiol* 2021;**52(1)**:195–206.

190. Schiavetto CM, de Abreu PM, von Zeidler SV, et al. Human Papillomavirus DNA Detection by Droplet Digital PCR in Formalin-Fixed Paraffin-Embedded Tumor Tissue from Oropharyngeal Squamous Cell Carcinoma Patients. *Mol Diagn Ther* 2021;**25(1)**:59–70.

191. de Abreu PM, Có ACG, Azevedo PL, et al. Frequency of HPV in oral cavity squamous cell carcinoma. *BMC Cancer*. 2018;**18(1)**:324.

192. Quintero K, Giraldo GA, Uribe ML , et al. Human papillomavirus types in cases of squamous cell carcinoma of head and neck in Colombia. *Braz J Otorhinolaryngol* 2013;**79(3)**:375–81.

193. Kaminagakura E, Villa LL, Andreoli MA, et al. High-risk human papillomavirus in oral squamous cell carcinoma of young patients. *Int J Cancer* 2012;**130(8)**:1726–32.

194. Anaya-Saavedra G, Ramírez-Amador V, Irigoyen-Camacho ME, et al. High Association of Human Papillomavirus Infection with Oral Cancer: A Case-Control Study. *Arch Med Res* 2008;**39(2)**:189–97.

195. Soares RCea. Detection of HPV DNA and immunohistochemical expression of cell cycle proteins in oral carcinoma in a population of brazilian patients. *J Appl Oral Sci* 2008;**16**:340–4.

196. da Silva CEXdSR, da Silva IDCG, Cerri A, Weckx LLM. Prevalence of human papillomavirus in squamous cell carcinoma of the tongue. *Oral Surg Oral Med Oral Pathol Oral Radiol Endodont* 2007;**104(4)**:497–500.

197. Rivero ER, Nunes FD. HPV in oral squamous cell carcinomas of a Brazilian population: amplification by PCR*. Braz Oral Res* 2006;**20(1)**:21–4.

198. Furrer VE, Benitez MB, Furnes M, Lanfranchi HE, Modesti NM. Biopsy vs. superficial scraping: detection of human papillomavirus 6, 11, 16, and 18 in potentially malignant and malignant oral lesions. *J Oral Pathol Med* 2006;**35(6)**:338–44.

199. Ibieta BR, Lizano M, Frı́as-Mendivil M, et al. Human papillomavirus in oral squamous cell carcinoma in a Mexican population. *Oral Surg Oral Med Oral Pathol Oral Radiol Endodont* 2005;**99(3)**:311–5.

200. Correnti M, Rivera H, Cavazza M. Detection of human papillomaviruses of high oncogenic potential in oral squamous cell carcinoma in a Venezuelan population. *Oral Dis* 2004;**10(3)**:163–6.

201. Premoli-De-Percoco G, Ramirez JL. High-risk human papillomavirus in oral squamous carcinoma: evidence of risk factors in a Venezuelan rural population. Preliminary report. *J Oral Pathol Med* 2001;**30(6)**:355–61.

202. Miguel RE, Villa LL, Cordeiro AC, Prado JC, Sobrinho JS, Kowalski LP. Low prevalence of human papillomavirus in a geographic region with a high incidence of head and neck cancer. *Am J Surg* 1998;**176(5)**:428–9.

203. Miller CS, Zeuss MS, White DK. Detection of HPV DNA in oral carcinoma using polymerase chain reaction together with in situ hybridization. *Oral Surg Oral Med Oral Pathol* 1994;**77(5)**:480–6.

204. Vanshika S, Preeti A, Sumaira Q, et al. Incidence of HPV and EBV in oral cancer and their clinico-pathological correlation- a pilot study of 108 cases. *J Oral Biol Craniofac Res* 2021;**11(2)**:180–4.

205. Hallikeri K, Burde K, Anehosur V, Kulkarni BB, Hiremath SV. p53 polymorphism and association of human papillomavirus in oral submucous fibrosis and oral squamous cell carcinoma: A case-control study. *J Oral Maxillofac Pathol* 2019;**23(1)**:97–103.

206. Sarmah N, Baruah MN, Baruah S. Immune Modulation in HLA-G Expressing Head and Neck Squamous Cell Carcinoma in Relation to Human Papilloma Virus Positivity: A Study From Northeast India. *Front Oncol* 2019;**9**:58.

207. Bandhary SK, Shetty V, Saldanha M, et al. Detection of Human PapillomaVirus and Risk Factors among Patients with Head and Neck Squamous Cell Carcinoma Attending a Tertiary Referral Centre in South India. *Asian Pac J Cancer Prev* 2018;**19(5)**:1325–30.

208. Dalakoti P, Ramaswamy B, Bhandarkar AM, Nayak DR, Sabeena S, Arunkumar G. Prevalence of HPV in Oral Squamous Cell Carcinoma in South West India. *Indian J Otolaryngol Head Neck Surg* 2019;**71(Suppl 1)**:657–64.

| 209. Grewal RK, Sircar K, Bhat KG, Grewal DS, Tyagi KK, David S. Detection of human papillomavirus-E6/E7 proteins of high-risk human papillomavirus in saliva and lesional tissue of oral squamous cell carcinoma patients using nested multiplex polymerase chain reaction: A comparative study. *J Oral Maxillofac Pathol* 2018;**22(3)**:318–24. |
| --- |

210. Chowdary SD, Sekhar PC, Kattapagari KK, Mani Deepthi CH, Neelima D, Reddy BVR. A study to assess expression of human papillomavirus types 16 and 18 in oral squamous cell carcinoma using polymerase chain reaction. *J Oral Maxillofac Pathol* 2018;**22(3)**:347–52.

211. Pal P, Raychowdhury R, Basu S, Gure PK, Das S, Halder A. Cytogenetic and micronuclei study of human papillomavirus-related oral squamous cell carcinoma. *J Oral Maxillofac Pathol* 2018;**22(3)**:335–40.

212. Palve V, Bagwan J, Krishnan NM, Pareek M, Chandola U, Suresh A, et al. Detection of High-Risk Human Papillomavirus in Oral Cavity Squamous Cell Carcinoma Using Multiple Analytes and Their Role in Patient Survival. *J Glob Oncol* 2018;**4**:1–33.

213. Pandey M, Kannepali KK, Dixit R, Kumar M. Effect of neoadjuvant chemotherapy and its correlation with HPV status, EGFR, Her-2-neu, and GADD45 expression in oral squamous cell carcinoma. *World J Surg Oncol* 2018;**16(1)**:20.

214. Kundu S, Ramshankar V, Verma AK, et al. Association of DFNA5, SYK, and NELL1 variants along with HPV infection in oral cancer among the prolonged tobacco-chewers. *Tumour Biol*. 2018 ;**40(8)**:1010428318793023.

215. Gheit T, Anantharaman D, Holzinger D, et al. Role of mucosal high-risk human papillomavirus types in head and neck cancers in central India. *Int J cancer* 2017;**141(1)**:143–51.

216. Verma G, Vishnoi K, Tyagi A, et al. Characterization of key transcription factors as molecular signatures of HPV-positive and HPV-negative oral cancers. *Cancer Med* 2017;**6(3)**:591–604.

217. Rajesh D, Mohiyuddin SMA, Kutty AVM, Balakrishna S. Prevalence of human papillomavirus in oral squamous cell carcinoma: A rural teaching hospital-based cross-sectional study. *Int J cancer* 2017;**54(3)**:498–501.

218. Pathak A, Singh M, Agarwal A, Amit S. Determination of p16 overexpression as an indicator of human papillomavirus infection in oral dysplasia and carcinoma. *Indian J Dent Res* 2017;**28(4)**:418–23.

219. Bhosale PG, Pandey M, Desai RS, et al. Low prevalence of transcriptionally active human papillomavirus in Indian patients with HNSCC and leukoplakia. *Oral Surg Oral Med Oral Pathol Oral Radiol* 2016;**122(5)**:609–18 e7.

220. Laprise C, Madathil SA, Allison P, et al. No role for human papillomavirus infection in oral cancers in a region in southern India. *Int J Cancer* 2016;**138(4)**:912–7.

221. Sannigrahi M, Singh V, Sharma R, Panda N, Radotra B, Khullar M. Detection of active human papillomavirus-16 in head and neck cancers of Asian North Indian patients. *Oral Dis* 2016;**22(1)**:62–8.

222. Murthy V, Swain M, Teni T, et al. Human papillomavirus/p16 positive head and neck cancer in India: Prevalence, clinical impact, and influence of tobacco use. *Indian J Cancer* 2016;**53(3)**:387–93.

223. Rathore AS, Gulati N, Shetty DC, Jain A. To analyze the concomitant expression of human papillomavirus-16 in the pathogenetic model of p53-dependant pathway in oral squamous cell carcinoma. *J Oral Maxillofac Pathol* 2016 S;**20(3)**:342–7.

224. Singh AK, Kushwaha JK, Anand A, et al. Human Papillomavirus in Oral Cavity Cancer and Relation to Change in Quality of Life Following Treatment—a Pilot Study from Northern India. *Indian J Surg Oncol* 2016;**7(4)**:386–91.

225. Bijina BR, Ahmed J, Shenoy N, Ongole R, Shenoy S, Baliga S. Detection of human papillomavirus in potentially malignant and malignant lesions of the oral cavity and a study of associated risk factors. *South Asian J Cancer* 2016;**5(4)**:179–81.

226. Dhanapal R, Ranganathan K, Kondaiah P, Devi RU, Joshua E, Saraswathi TR. High-risk human papillomavirus in archival tissues of oral pathosis and normal oral mucosa. *Contemp Clin Dent* 2015;**6(2)**:148–52.

227. Parshad S, Nandi S, Marwah N, et al. Human papillomavirus 16 and 18 in squamous cell carcinoma of oral cavity and sexual practices: A pilot study at a Tertiary Care Hospital of North India. *Natl J Maxillofac Surg* 2015;**6(2)**:185–9.

228. Singh V, Husain N, Akhtar N, et al. Do Human Papilloma Viruses Play Any Role in Oral Squamous Cell Carcinoma in North Indians? *Asian Pac J Cancer Prev* 2015;**16**:7077–84.

229. Krishnan N, Gupta S, Palve V, et al. Integrated analysis of oral tongue squamous cell carcinoma identifies key variants and pathways linked to risk habits, HPV, clinical parameters and tumor recurrence. *F1000Research* 2015;**4**:1215.

230. Kumar R RA, Das D. Alcohol and Tobacco increases Risk of High-Risk HPV Infection in Head and Neck Cancer Patients: Study from North-East Region of India. *PloS One* 2015;**10**:e0140700.

231. Talukdar FR, Ghosh SK, Laskar RS, Kannan R, Choudhury B, Bhowmik A. Epigenetic pathogenesis of human papillomavirus in upper aerodigestive tract cancers.
*Mol Carcinog* 2015;**54(11)**:1387–96.

232. Patel KR, Vajaria BN, Begum R, et al. Prevalence of high-risk human papillomavirus type 16 and 18 in oral and cervical cancers in population from Gujarat, West India. *J Oral Pathol Med* 2014;**43(4)**:293–7.

233. Ramshankar V, Soundara VT, Shyamsundar V, Ramani P, Krishnamurthy A. Risk stratification of early stage oral tongue cancers based on HPV status and p16 immunoexpression. *Asian Pac J Cancer Prev* 2014;**15(19)**:8351–9.

| 234. Chakrobarty B, Roy JG, Majumdar S, Uppala D. Relationship among tobacco habits, human papillomavirus (HPV) infection, p53 polymorphism/mutation and the risk of oral squamous cell carcinoma. *J Oral Maxillofac Pathol* 2014;**18(2)**:211–6. |
| --- |

235. Maitra A, Biswas NK, Amin K, et al. Mutational landscape of gingivo-buccal oral squamous cell carcinoma reveals new recurrently-mutated genes and molecular subgroups. *Nat Commun* 2013;**4(1)**:2873.

236. Bahl A, Kumar P, Dar L, et al. Prevalence and trends of human papillomavirus in oropharyngeal cancer in a predominantly north Indian population. *Head Neck* 2014;**36(4)**:505–10.

237. Kulkarni SS, Kulkarni SS, Vastrad PP, et al. Prevalence and distribution of high risk human papillomavirus (HPV) Types 16 and 18 in Carcinoma of cervix, saliva of patients with oral squamous cell carcinoma and in the general population in Karnataka, India. *Asian Pac J Cancer Prev* 2011;**12(3)**:645–8.

238. Elango KJ, Suresh A, Erode EM, et al. Role of human papillomavirus in oral tongue squamous cell carcinoma. *Asian Pac J Cancer Prev* 2011;**12(4)**:889–96.

239. Barwad A, Sood S, Gupta N, Rajwanshi A, Panda N, Srinivasan R. Human papillomavirus associated head and neck cancer: A PCR based study. *Diagn Cytopathol* 2012;**40(10)**:893–7.

240. Chaudhary AK, Pandya S, Mehrotra R, Bharti AC, Singh M, Singh M. Comparative study between the Hybrid Capture II test and PCR-based assay for the detection of human papillomavirus DNA in oral submucous fibrosis and oral squamous cell carcinoma. *Virol J* 2010;**7(1)**:253.

241. Jalouli J, Ibrahim SO, Mehrotra R, et al. Prevalence of viral (HPV, EBV, HSV) infections in oral submucous fibrosis and oral cancer from India.
*Acta Otolaryngol* 2010;**130(11)**:1306–11.

242. Bhattacharya N, Roy A, Roy B, Roychoudhury S, Panda CK. MYC gene amplification reveals clinical association with head and neck squamous cell carcinoma in Indian patients. *J Oral Pathol Med* 2009;**38(10)**:759–63.

243. Majumder M, Indra D, Roy PD, et al. Variant haplotypes at XRCC1 and risk of oral leukoplakia in HPV non-infected samples. *J Oral Pathol Med* 2009;**38(2)**:174–80.

244. Ghosh A, Ghosh S, Maiti GP, et al. SH3GL2 and CDKN2A/2B loci are independently altered in early dysplastic lesions of head and neck: correlation with HPV infection and tobacco habit. *J Pathol* 2009;**217(3)**:408–19.

245. Gheit T, Vaccarella S, Schmitt M, et al. Prevalence of human papillomavirus types in cervical and oral cancers in central India. *Vaccine* 2009;**27(5)**:636–9.

246. Mitra S, Banerjee S, Misra C, et al. Interplay between human papillomavirus infection and p53 gene alterations in head and neck squamous cell carcinoma of an Indian patient population. *J Clin Pathol* 2007;**60(9)**:1040–7.

247. Mishra A, Bharti AC, Varghese P, Saluja D, Das BC. Differential expression and activation of NF-kappaB family proteins during oral carcinogenesis: Role of high-risk human papillomavirus infection. *Int J Cancer* 2006;**119(12)**:2840–50.

248. Koppikar P, deVilliers E-M, Mulherkar R. Identification of human papillomaviruses in tumors of the oral cavity in an Indian community. *Int J Cancer* 2005;**113(6)**:946–50.

249. Katiyar S, Thelma BK, Murthy NS, et al. Polymorphism of the p53 codon 72 Arg/Pro and the risk of HPV type 16/18-associated cervical and oral cancer in India. *Mol Cell Biochem* 2003;**252(1**–**2)**:117–24.

250. Herrero R, Castellsagué X, Pawlita M, et al. Human papillomavirus and oral cancer: the International Agency for Research on Cancer multicenter study. *J Natl Cancer Inst* 2003;**95(23)**:1772–83.

251. Nagpal JK, Patnaik S, Das BR. Prevalence of high-risk human papillomavirus types and its association with P53 codon 72 polymorphism in tobacco addicted oral squamous cell carcinoma (OSCC) patients of Eastern India. *Int J Cancer* 2002;**97(5)**:649–53.

252. D'Costa J, Saranath D, Dedhia P, Sanghvi V, Mehta AR. Detection of HPV-16 genome in human oral cancers and potentially malignant lesions from India. *Oral Oncol* 1998;**34(5)**:413–20.

253. Balaram P, Nalinakumar KR, Abraham E, et al. Human papillomaviruses in 91 oral cancers from indian betel quid chewers—high prevalence and multiplicity of infections. *Int J Cancer* 1995;**61(4)**:450–4.
